# The Synergistic Effect of Combining Electron Transfer and Photoactivation in Hydroxyapatite/ZrO_2_ Nanocomposites Promotes Efficient Nitrogen-to-Ammonia Fixation Reaction

**DOI:** 10.1021/acscatal.5c02426

**Published:** 2025-06-12

**Authors:** Marc Arnau, Lukas Pielsticker, Walid Hetaba, Jordi Casanovas, Pau Turon, Carlos Alemán, Jordi Sans

**Affiliations:** † Departament d’Enginyeria Química, EEBE, 16767 Universitat Politècnica de Catalunya, C/Eduard Maristany, 10-14, Ed. I2, 08019, Barcelona, Spain; ‡ Barcelona Research Center in Multiscale Science and Engineering, Universitat Politècnica de Catalunya, C/Eduard Maristany, 10-14, 08019, Barcelona, Spain; § Department of Heterogeneous Reactions, Max Planck Institute for Chemical Energy Conversion, Stiftstr. 34-36, 45470, Muelheim an der Ruhr, Germany; ∥ Departament de Química, Física i Ciències Ambientals i del Sòl, 16739 Universitat de Lleida Escola Politècnica Superior, C/Jaume II n°69, Lleida E-25001, Spain; ⊥ B. Braun Surgical, S.A.U., Carretera de Terrassa 121, 08191 Rubí (Barcelona), Spain; # Institute for Bioengineering of Catalonia (IBEC), The Barcelona Institute of Science and Technology, Baldiri Reixac 10-12, 08028 Barcelona Spain

**Keywords:** Hydroxyapatite, Green Catalysis, Electron Enhanced Properties, π-Back-donation, Permanently Polarized Materials

## Abstract

Catalytically active hydroxyapatite (ca-HAp) decorated with zirconia nanoparticles (ZrO_2_ NPs) is presented as a nanocomposite catalyst (ca-HAp/ZrO_2_) capable of performing highly efficient nitrogen to ammonia (N_2_-to-NH_3_) fixation reactions under mild conditions. Accordingly, reactions were carried out in a batch reactor operating at 120 °C, 6 bar of N_2_, and 20 mL of water, under UV irradiation (14 W) for 72 h. The yield of NH_3_ obtained was 1.592 ± 0.146 mmol·g_c_^–1^, which represents a N_2_ fixation efficiency of 6.4%. Near ambient pressure X-ray photoelectron spectroscopy (NAP-XPS) studies under *in situ* conditions (i.e., at elevated pressure and temperature and during UV irradiation) and density functional theory simulations (DFT) allowed us to elucidate the catalytic mechanism of the system. The ca-HAp/ZrO_2_ nanocomposites exhibit a strong synergy arising from the initial photoactivation of N_2_ by means of the π-backdonation mechanism in ZrO_2_ (N_2_ is anchored by four Zr^4+^ atoms) followed by the dinitrogen spillover toward the Ca­(I)^2+^ binding sites. Such sites, preferentially exposed in the (001) crystallographic planes of ca-HAp, show high activity due to the enhanced electron transfer properties of ca-HAp. These catalytic nanocomposites represent a viable alternative to the conventional catalysts used for N_2_-to-NH_3_ fixation reactions.

## Graphical abstract

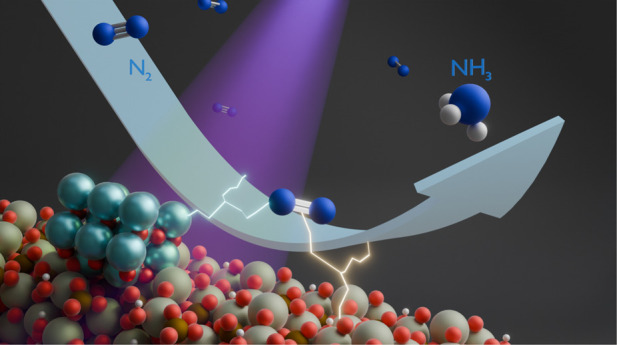

## Introduction

Within the ambitious framework of net-zero emissions adopted by most developed countries by 2050, the industrial nitrogen (N_2_)-to-ammonia (NH_3_) synthesis reaction through the Haber–Bosch (H–B) process has been put in the spotlight due to its high environmental impact. The H–B process is responsible for 1–2% of the global carbon dioxide (CO_2_) emissions^1,2^ due to both the extreme reaction conditions (300–500 °C and 150–300 atm) needed for the N_2_ fixation and the use of H_2_ gas (produced by the steam reforming of methane).^1,3^ Besides, rather than decreasing, the NH_3_ demands are expected to increase in the 21st century, as apart from being an essential compound to sustain food, it also represents a promising hydrogen energy carrier.^4^ Hence, a complete transition toward sustainable N_2_-to-NH_3_ fixation is crucial to avoid several well-known decarbonization paradoxes (i.e., a final positive CO_2_ emission balance is obtained when considering the overall energetic costs).^5,6^ Such transition not only faces the challenging reduction of the energetic requirements for the N_2_ triple-bond cleavage (945 kJ·mol^–1^), reducing also the complexity of the installations needed, but also should focus on (1) replacing H_2_ as the proton source, ideally by other more available hydrogen donor compounds such as water,^7^ and (2) promoting the use of green catalysts with lower environmental impact instead of conventional metallic (i.e., use of rare-earth and heavy metals) catalysts, which also present considerably higher costs.^8^

In the recent years, significant progress has been made toward green N_2_-to-NH_3_ fixation through photo and photoelectrochemical catalysis motivated by the favorable energetic balance derived from solar energy.^9–14^ Accordingly, the possibility to carry out N_2_-to-NH_3_ reactions at <200 °C merits special attention.^15–17^ One of the most promising strategies relies on the N_2_ activation through the π-backdonation mechanism (i.e., transfer of 2e^–^ from the d orbital of the catalyst to the π* antibonding orbital of N_2_).^18–21^ Nonetheless, the efficiency of such an approach is restricted by the lack of appropriate and sufficient active binding sites, which can also promote hydrogen evolution reactions in electrocatalysis (low Faradaic efficiencies due to saturation of the binding sites)^22^ or favor electron–hole recombination in photocatalysis (due to the poor N_2_ adsorption on conventional metal-oxide photocatalysts).^11^ Therefore, beyond the conventional exploration of the catalytic activity of other elements (specifically, rare-earth metals), it is crucial to develop catalyst synergies that enhance electron transfer and increase the number of available binding centers. Indeed, several strategies have been reported to improve N_2_-to-NH_3_ yields under mild conditions such as doping of nanostructured materials,^23–25^ vacancy engineering,^26–29^ or heteronuclear metal dual-site catalysts and cocatalysts,^30–32^ among others.^33,34^

Unfortunately, most reported studies cannot yet be considered truly sustainable N_2_-to-NH_3_ reactions due to the non-completely green nature of the catalysts and/or electrolytes used. Interestingly, Légaré et al. reported the N_2_ activation by means of nonmetallic boron.^35^ The mechanism reported relies on the use of sp^3^ orbitals for similar π-backdonation N_2_ activation, which has been further explored for graphitic carbon nitride^36^ (among other more complex heteronuclear metal-free double-atom catalysis approaches),^31^ noncritical carbon materials,^37^ or Si-based complexes,^38^ showing excellent activity. On the other hand, we have recently proposed hydroxyapatite (HAp, Ca_5_(PO_4_)_3_OH) as a naturally abundant, green, and biocompatible catalyst (HAp is the main constituent of hard tissues), capable of conducting carbon and nitrogen fixation reactions under mild conditions (95 °C, 1–6 bar, UV irradiation) and with water as the hydrogen source.^39–43^ Investigations on such catalytically active HAp (ca-HAp) have been mainly focused on increasing the exposed surface area,^44,45^ improving the density of CO_2_ binding centers and electronic properties for simultaneous water splitting and carbon fixation (vacancy engineering and lattice refinement through a permanent polarization process, TSP),^46–48^ or providing superior synergies with nanoparticles (generation of a p–n junction nanocomposite favoring electron–hole separation),^49^ leading to increased yield and selectivity in CO_2_ fixation reactions. Conversely, the sustainable N_2_-to-NH_3_ fixation catalyzed by ca-HAp has been scarcely explored.^41^ Consequently, the specific role of ca-HAp binding sites remains uncertain, and more importantly, the N_2_ activation has not been properly assessed, critically hindering its efficiency toward such reaction.

Herein, we present a novel and green ca-HAp/ZrO_2_ nanocomposite for the sustainable N_2_-to-NH_3_ fixation reaction, showing boosted efficiency. Both materials have been carefully chosen considering their high natural abundances, high thermal stability (due to the TSP conditions), and biocompatible properties, ensuring this way the true green nature of the catalytic system reported. More specifically, we explore the synergies of coupling-optimized electron transfer (ca-HAp) and photoactivation of N_2_ by means of the π-backdonation approach (ZrO_2_). To do so, near-ambient-pressure X-ray photoelectron spectroscopy (NAP-XPS) measurements under *in situ* conditions (i.e., at elevated pressure and temperature and during UV irradiation) supported by density functional theory (DFT) calculations have been carried out, providing essential mechanistic insights about the performance of the catalyst for further optimization.

## Results

### Design of the Catalyst Nanocomposite

Because of the novelty of using a ca-HAp/ZrO_2_ nanocomposite as a catalyst for N_2_-to-NH_3_, the fundamental catalytic strategies adopted are presented below. Due to its singular chemical composition and crystallographic structure, HAp has both acid Lewis sites (Ca^2+^) and basic sites (PO_4_^3–^ and OH^–^).^50^ As depicted in Figure 1a, vacancy engineering is considered to increase the activity and density of binding sites by introducing OH^–^ vacancies through a thermal treatment (optimum temperature between 900 and 1100 °C).^46^ Moreover, the process also stabilizes the HAp structure and increases both the crystallinity and specific surface charge of HAp (Figures S1–S3). The second step to obtain ca-HAp (presented in Figure 1b) relies on imposing a specific orientation to the remaining OH^–^ groups, which in their pristine hexagonal *P*6_3_/*m* form are aligned in columns along the *c*-axis but without pointing toward any specific direction. To do so, the thermal stimulated polarization (TSP) treatment is applied, resulting in its optimized conditions (i.e., 1000 °C and 500 V), in a permanently polarized state.^47^ Consequently, the OH^–^ columns act as quantum electronic pathways, showing enhanced proton conductivity and charge delocalization (Figure S4). Accordingly, the activity of the aforementioned binding sites is enhanced, resulting in increased catalytic activation of HAp. Moreover, the TSP treatment results in superior crystallinity and the controlled generation of a pseudo-brushite phase (CaHPO_4_), as observed by X-ray diffraction (XRD) and Raman spectroscopy (Figures S5 and S6). This phase not only is responsible for the plasticity properties of ca-HAp^40^ but also introduces more acid sites (i.e., P–OH Brønsted irreversible adsorption sites).^50^ Additionally, HAp can be initially shaped into porous cubes (Figure 1c) to increase the catalytic surface area without restricting the effect of the TSP treatment applied afterward.^44^ Overall, ca-HAp has been designed to show a high density of binding sites and to favor the evolution of nitrogen intermediates toward NH_3_ due to its enhanced electronic properties.

**Figure 1:**
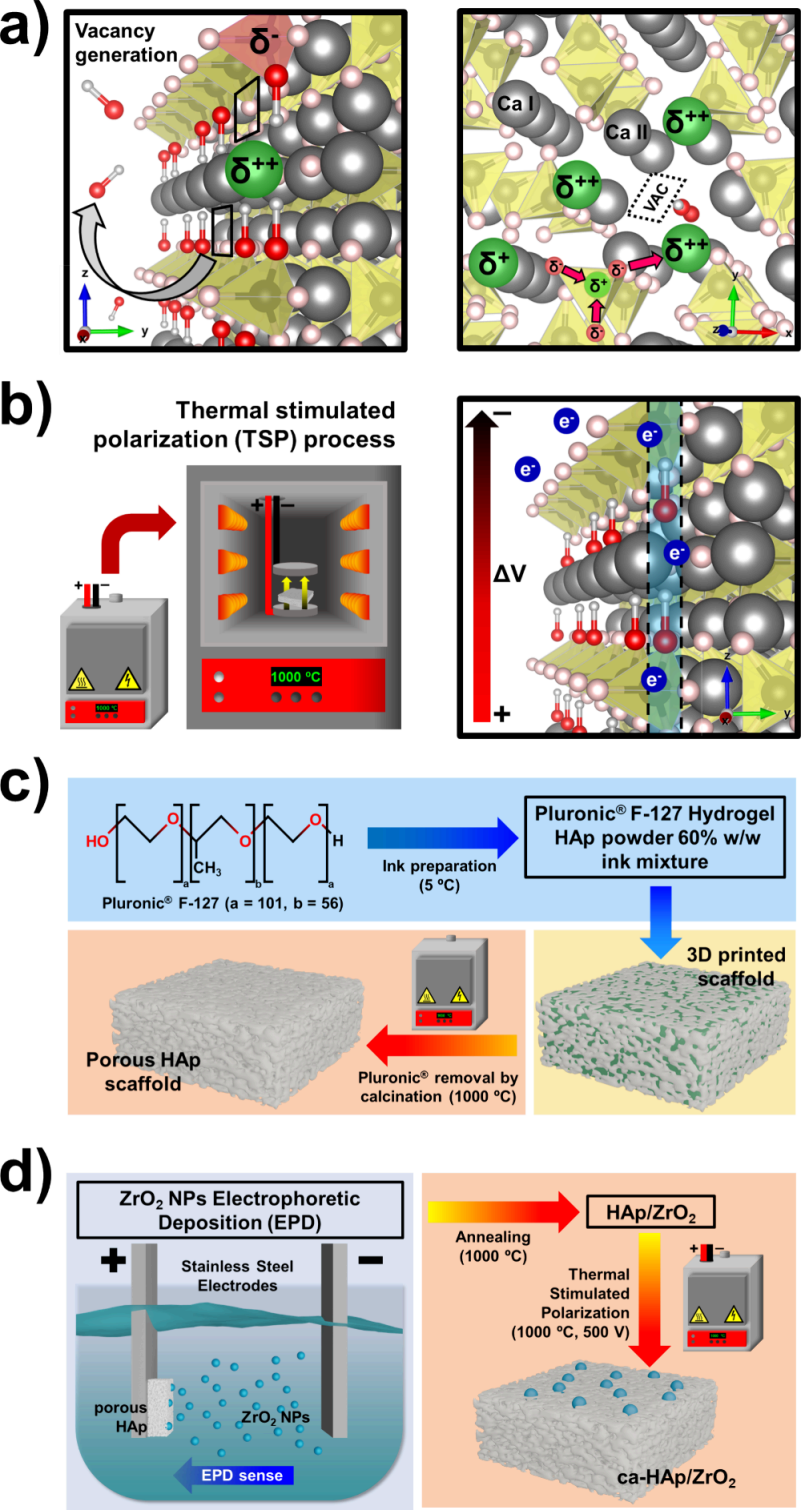
Scheme of the design and synthesis of the ca-HAp/ZrO_2_ nanocomposites. (a) Structural sketch of the OH^–^ vacancy generation achieved by submitting HAp to a sintering treatment (1000 °C). The crystallographic positions of the acid and basic binding sites (δ, green and red; respectively), as well as the OH^–^ vacancies (“*VAC”* in black rectangles), are highlighted. (b) Experimental setup used for the thermal polarization process (left). Resultant lattice structure (right), showing the remaining OH^–^ pointing in the direction of the electrical field applied. The presence of the electronic quantum pathways responsible for the enhanced electrical properties of ca-HAp is also displayed. (c) Scheme of the preparation of the ultraporous HAp cubes using Pluronic F-127 as a sacrificial agent. (d) ZrO_2_ NP loading by the electrophoretic deposition process on the ultraporous HAp cubes (left) and the final catalytic activation (i.e., TSP treatment) of the whole ca-HAp/ZrO_2_ nanocomposite (right).

On the other hand, ZrO_2_ is deposited as nanoparticles (NPs) onto the surface of HAp porous cubes to promote the photoactivation of N_2_ by means of the π-backdonation mechanism, but without restricting the adsorption of N_2_ onto the ca-HAp surface. For this reason, the deposition of ZrO_2_ NPs has been carried out through electrophoresis (Figure 1d), as it offers high control of the deposition process compared with other techniques such as drop casting or dry impregnation. Preliminary studies and optimization of the experimental parameters (e.g., voltage applied or electrolyte used) are shown in Figures S7 and S8. According to previous works,^49^ the TSP treatment has been applied to the whole composite in order to not only activate HAp (ca-HAp) but also enhance the potential synergistic effects between ca-HAp and ZrO_2_. Note the change in the paradigm involved in this study, where apart from being a mechanical support for ZrO_2_ NPs, ca-HAp is used as an active catalytic support, as it also provides N_2_ binding centers.

### Structural Characterization of the ca-HAp/ZrO_2_ Nanocomposites

The successful incorporation of ∼90 nm ZrO_2_ NPs onto the HAp carved surface in ca-HAp/ZrO_2_ nanocomposites has been confirmed through electron microscopy in Figures 2a–i. The high-resolution transmission electron microscopy (HRTEM) images presented in parts a and e reveal the well-resolved and distinctive lattice fringes of highly crystalline HAp (red) and ZrO_2_ NPs (blue). According to the corresponding Fourier transformations (shown as insets), the tetragonal ZrO_2_ NPs (*P*4_2_/*nmc*, see Figure S9 for polymorph determination) present the characteristic (001), (002), (110), (101), and (102) crystallographic planes at 5.24, 2.65, 2.56, 2.95, and 2.11 Å, respectively, while the characteristic crystallographic planes for the HAp (*P*6_3_/*m*) are found at 2.75 Å (112), 2.82 Å (121), 3.16 Å (102), 3.41 Å (002), and 3.50 Å (201). Moreover, the permanent effect of the TSP treatment on HAp has been attributed to the existence of the characteristic superstructure observed at 6.76 Å (001), which is assumed to stabilize the resulting ca-HAp lattice.^48^ Special attention has been put on carefully examining the grain boundary between the ZrO_2_ NPs and the ca-HAp (Figure 2e, image acquired at higher magnification) surface due to its importance in boosting electron transfer as far as avoiding electron–hole recombination phenomena. As it can be seen by optical inspection, both crystallographic lattices merge smoothly without introducing any relevant distortion, ensuring optimum electronic synergies. According to the literature, such behavior is promoted by applying the TSP treatment to the overall nanocomposite.^49^

**Figure 2:**
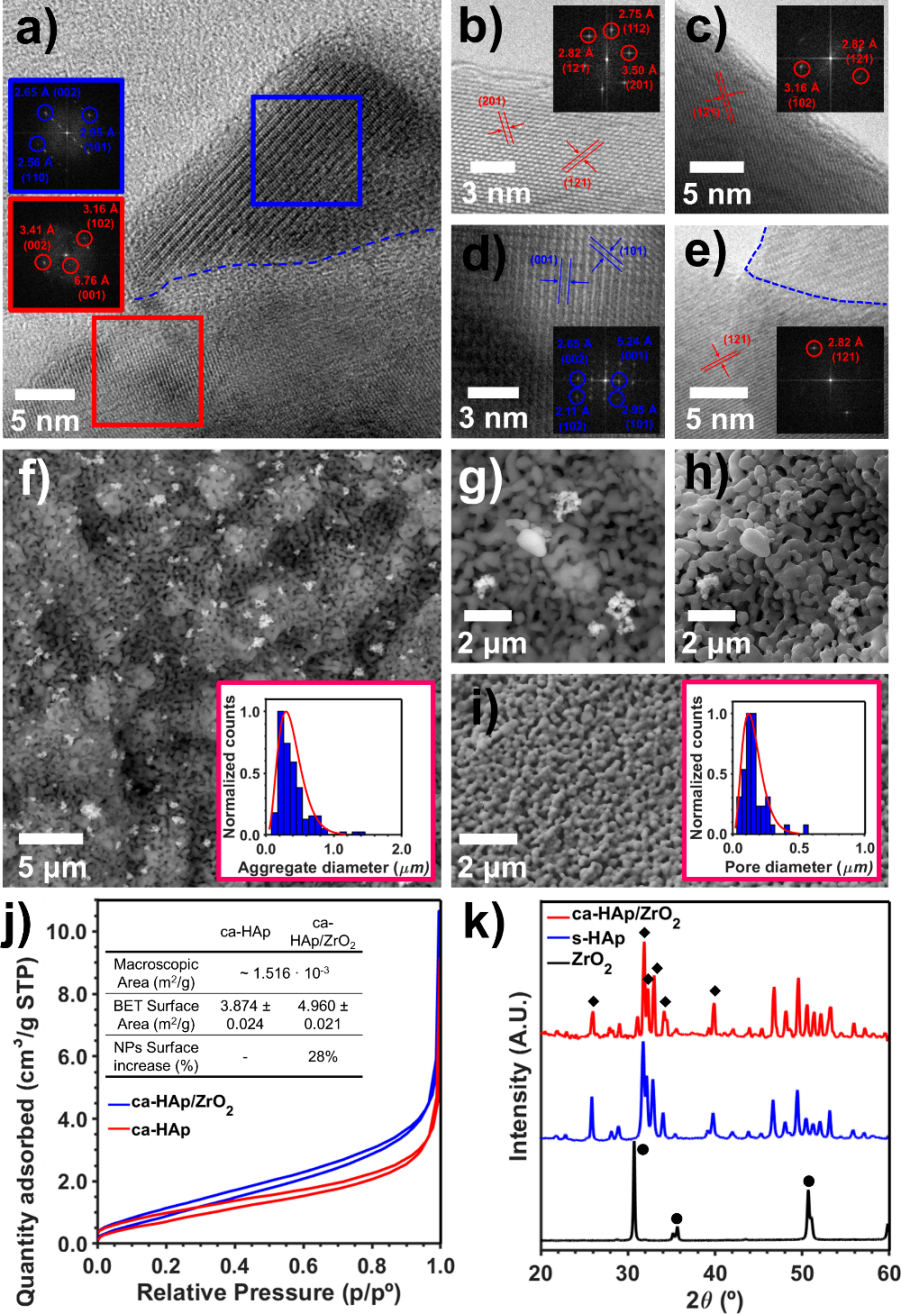
Structural and morphological characterization of the ca-HAp/ZrO_2_ nanocomposites. (a–e) HRTEM images with the corresponding Fourier transformations of ca-HAp (red) and tetragonal ZrO_2_ (blue). The interface between ca-HAp and the ZrO_2_ NPs is marked with a dashed blue line. (f) SEM image acquired using the backscattered electron detector. The distribution of the ZrO_2_ aggregates is shown in the inset. (g, h) SEM images acquired with the backscattered electron detector (g) and secondary electron detector (h). (i) SEM images of the ultraporous HAp cubes (secondary electron detector). The pore diameter distribution is depicted in the inset. (j) BET studies comparing ca-HAp (used as reference) and ca-HAp/ZrO_2_ samples. (k) XRD pattern obtained for ZrO_2_ NPs, sintered HAp (s-HAp), and ca-HAp/ZrO_2_ samples. The characteristic reflections of tetragonal ZrO_2_ and HAp are marked with circles and diamonds, respectively.

The scanning electron microscopy (SEM) images acquired using both the backscattered electron (Figures 2f and 2g) and the secondary electron (Figures 2h and 2i) detectors reveal the presence of small ZrO_2_ NP aggregates of 384 ± 216 nm (see inset in Figure 2f) deposited onto a porous ca-HAp surface (pore size of 158 ± 93 nm, inset of Figure 2i). Accordingly, the loading of the ZrO_2_ NPs results in an increment of 28% of the total surface, which was determined by the Brunauer–Emmet–Teller (BET) surface area analysis as shown in Figure 2j (3.874 ± 0.024 m^2^/g and 4.960 ± 0.021 m^2^/g for ca-HAp and ca-HAp/ZrO_2_, respectively). The characterization of the ca-HAp/ZrO_2_ cross-section in Figure S10 discards the presence of additional ZrO_2_ NPs inside the bulk of ca-HAp. Nonetheless, Figure S10 reveals that the high porosity observed at the surface is also maintained throughout the whole bulk. Finally, the XRD patterns in Figure 2k discard the presence of bigger ZrO_2_ agglomerates, as only the characteristic diffraction peaks of HAp (black diamonds) can be observed in the nanocomposite.

### N_2_-to-NH_3_ Fixation Reaction

The N_2_-to-NH_3_ fixation reactions were carried out in a batch reactor (Figure S11) at 120 °C, 6 bar of N_2_, and UV irradiation for 72 h. Additionally, 20 mL of H_2_O (used as the proton source) was loaded into the reactor. As proposed by R. Shi and co-workers, we do not use any sacrificial agent (apart from water), relying on the specific design of the catalytic system to enhance the catalytic yields.^51^ After each reaction, the liquid remaining in the reactor was collected to analyze the products obtained by means of ^1^H NMR. The corresponding blank measurements are provided in Figure S12. Figure 3a presents the yields of the N_2_-to-NH_3_ reaction obtained by ca-HAp/ZrO_2_ in front of other catalytic systems considering ca-HAp and ca-ZrO_2_ (i.e., ZrO_2_ sample that has been exposed to the TSP treatment) separately, which serve as controls, and nonpolarized HAp/ZrO_2_. The characteristic ^1^H NMR triplet (1:1:1) of NH_4_^+^ at 7.030, 6.943, and 6.856 ppm has been used for quantification (Figure 3b). The production of NH_3_ was further corroborated through isotopic ^15^N_2_-to-^15^NH_3_ reactions, as ^15^NH_4_^+^ results in a doublet at 7.19 and 7.00 ppm (1:1) due to the change in nuclear spin (*s* = 1 and 1/2 for ^14^N and ^15^N, respectively).^52^ The representative spectra derived from the isotopic experiments are depicted in Figure 3c.

**Figure 3:**
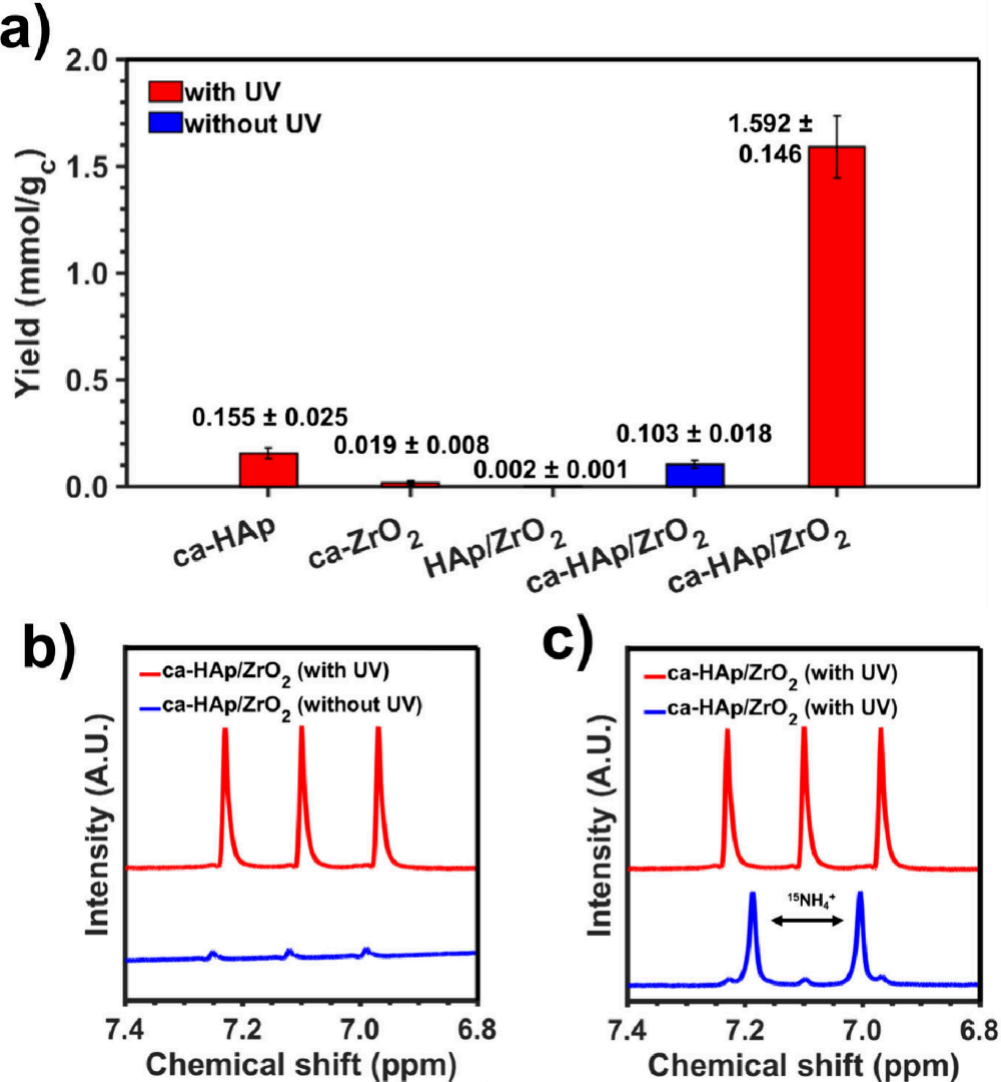
(a) Yield of ammonia (mmol of ammonia per gram of catalyst) obtained using different catalysts. The reactions were carried out at 120 °C, 6 bar of N_2_ with 20 mL of water, for 72 h and with (red) or without (blue) exposure of UV light (14 W, λ = 253.7 nm). (b) Dependence on the UV light irradiation. The characteristic ^1^H NMR ^14^NH_4_^+^ triplet has been used for quantification. (c) ^1^H NMR spectra obtained for isotope labeled experiments: ^14^N_2_-to-^14^NH_3_ (red) and ^15^N_2_-to-^15^NH_3_ (blue).

As can be observed in Figure 3a, ca-HAp presents a remarkable higher production of NH_3_ per gram of catalyst (0.155 ± 0.025 mmol/g_c_) compared with sintered HAp (0.002 ± 0.001 mmol/g_c_) and ZrO_2_ after applying the TSP treatment (ca-ZrO_2_, 0.019 ± 0.008 mmol/g_c_), highlighting its active catalytic role. A strong synergy appears for ca-HAp/ZrO_2_, as the catalytic yield obtained increases by a factor of ∼900%, achieving the production of 1.592 ± 0.146 mmol/g_c_. Although the study of the effect of the temperature presented in Figure S13 confirms the positive production of ammonia at 80 °C (179.60 ± 4.49 μmol/g_c_), results show that temperatures slightly higher than the water boiling point are necessary to boost the efficiency of the reaction. We attribute this behavior to the increase of water vapor pressure^53^ governed by the Antoine equation,^54^ which shows the same trend as the yields in Figure S13. Hence, apart from the direct energetic contribution, temperature is also needed to achieve highly saturated water atmospheres acting as proton sources, being in complete agreement with the rate-limiting steps determined for other reactions catalyzed by ca-HAp.^55^ As it can be seen, the photoactivation of N_2_ assisted by UV irradiation becomes essential to reach the high yields observed (the yield for ca-HAp/ZrO_2_ without UV irradiation is just 0.103 ± 0.018 mmol/g_c_). Interestingly, the N_2_-to-NH_3_ positive reaction in the dark is only driven by ca-HAp/ZrO_2_ (further validated through more repetitions). Besides, extra reactions carried out reveal that ca-HAp and HAp/ZrO_2_ systems do not show any ammonia production without irradiation, thus highlighting the unique tandem formed by ca-HAp and ZrO_2_ that promotes charge separation and charge transference. Similar electronic mechanisms have been proposed for ca-HAp decorated with TiO_2_ NPs.^49^ Furthermore, such feature is in agreement with the results reported by Y. Peng and co-workers that, using other catalytic systems supported on ZrO_2_, proposed that light-assisted and thermal reactions could share the same limiting steps in the reaction mechanism, thus reporting the successful N_2_-to-NH_3_ reaction under dark conditions.^56^ Accordingly, UV–vis spectra measured for both ca-ZrO_2_ and ca-HAp show a wide adsorption band at ∼215 nm (Figure S14). Moreover, the shoulder at 361 nm extends the total adsorption range toward UVB and UVA regions. Such a feature merits special relevance for sustainable N_2_-to-NH_3_ reactions due to the possibility of using direct sunlight as the irradiation source and thermal source. The stability of the catalyst has been assessed through characterization of the ca-HAp/ZrO_2_ nanocomposites after the reaction (Figure S15) and through a series of 5 consecutive 72 h reactions (standard conditions) using the same catalyst. The results highlight the proper stability of the system, as the ammonia yields obtained do not differ more than ∼10% (i.e., ±0.157 mmol/g_c_) and do not show any specific trend: a value close to the experimental error of the replicas.

In order to understand the impact and advantages of novel catalysts (i.e., ca-HAp/ZrO_2_), a N_2_-to-NH_3_ yield comparison is usually established in the literature. However, such comparisons are often complex and hinder the relevance of specific works due to a high variety of factors that affect the final yield value such as the reaction conditions, setup used (continuous or batch reaction), or yield normalization (per gram of catalyst or per surface area of catalyst), among others. In particular, when focusing on green N_2_-to-NH_3_ reactions considering the catalyst abundance, the electrolyte/solvent used and the final energetic balance become essential.

In this sense, it is worth mentioning that although ca-HAp/ZrO_2_ strongly relies on electron charge transport mechanisms (similar to electrocatalysts), ca-HAp/ZrO_2_ is foremost considered as a photothermal catalyst, as neither external electrical currents nor acid electrolytes are needed. Moreover, the abundance of all components of ca-HAp/ZrO_2_ is extremely high (i.e., Ca fifth, P 12th, and Zr 18th highest abundance in Earth’s crust) compared with rare earth elements and precious metals used in conventional electrocatalysts. Anyhow and assuming all the energetic drawbacks, the N_2_-to-NH_3_ yield obtained with ca-HAp/ZrO_2_ (∼22.11 μmol·g_c_^–1^·h^–1^, considering the theoretical 72 h batch reaction) is only 1 to 2 orders of magnitude lower as compared with the current electrocatalysts studied.^22^ On the other hand, the relevance of ca-HAp/ZrO_2_ becomes more obvious when considering the reaction conditions used for the photocatalytic approach. Although the high difference in catalyst abundances is maintained, with the use of different reagents (H_2_, Na_2_SO_4_) and/or hole scavengers (methanol, glycerol, triethanolamine), the yields obtained are found in the same range of the present study (i.e., several micromoles to a few hundred micromoles per gram of catalyst per hour).^11,14,34^ That is because in most of the studies reported,^11^ the power of the lamp used (300–500 W) is 21–36 times greater than the lamp used in this work (14 W).

Alternatively, the N_2_ fixation efficiency can also be used as a parameter to analyze the quality of a catalytic system. For ca-HAp/ZrO_2_ the N_2_ fixation efficiency is 6.4%, showing great potential for industrial applications. Certainly, the H–B reactions (thermocatalytic approach) catalyzed by an Fe/Ru-based catalyst at 200–300 atm (using N_2_ and H_2_) present an efficiency of ∼20%.^11^ Furthermore, hydrogenation reactions at similar mild conditions (150 °C and 9.9 atm N_2_:H_2_ = 1:3) catalyzed by Fe-LiH result in the ammonia production of 69 μmol·g_c_^–1^·h^–1^.^57^ Anyhow, such approaches rely on the use of H_2_, while our study uses water as the proton source. On the other hand, we have not found ZrO_2_ systems as solely photocatalysts for N_2_-to-NH_3_.^11,14,34,58^ Most similar catalysts (considering the green nature and sustainability) are oxygen-deficient titanium oxide (TiO_2_-OV) based catalysts showing a production of 0.76 μmol·g_c_^–1^·h^–1^ alone or 78.6 μmol·g_c_^–1^·h^–1^ when decorated with gold NPs, even though methanol is needed as a sacrificial agent.^11,14^ Overall, the strong synergy arising from electrothermal catalytic activity of ca-HAp and the photo contribution of ZrO_2_ is demonstrated as a potential strategy for the design of novel and green catalysts. Certainly, such a result postulates ca-HAp/ZrO_2_ as a direct viable green alternative to conventional photo- and electrocatalysts.

### Mechanistic Insights through NAP-XPS Measurements

*In situ* NAP-XPS studies were carried out to gain further mechanistic insights into the system. N_2_ gas was introduced into the reaction chamber until the pressure reached 1.0 mbar. To mimic the UV irradiation in the reaction chamber, an ultraviolet photoelectron spectroscopy (UPS) source, which is typically used for near-ambient pressure UPS (NAP-UPS) experiments, was used to irradiate the sample surface. Measurements were performed at 25, 95, and 120 °C, respectively, with the UPS source turned on and off, in order to understand the effect that the UV light has on the surface chemical state of the catalyst under reaction conditions. Certainly, the measurements under UV irradiation merit special attention as a quite novel approach for catalyst characterization. Herein, the spectra have been acquired using the UPS source (UV irradiation) operating simultaneously with the XPS source (sensing). Interestingly, the charging effects observed in ultra-high-vacuum (UHV) conditions were fully compensated when the sample was exposed to UV irradiation. This phenomenon has been only recently investigated by de los Arcos et al.^59^ and was attributed to the higher electron density due to the larger cross section interaction of UV irradiation with the gas, as compared with X-rays.

High-resolution NAP-XPS spectra of Ca 2p, P 2p, and O 1s of ca-HAp under different conditions (e.g., presence of N_2_ gas, temperature, and UV irradiation) are presented in Figures 4a–c, respectively. As soon as the reaction conditions are reached, new species of Ca^2+^ can be clearly observed at 348.8 eV for Ca 2p_3/2_ and 352.4 eV for Ca 2p_1/2_ that shifted 2.0 eV from their original oxidation state (Figures 4a and 4h). Such a shift is attributed to the anchoring of activated nitrogen species (*N_2_) onto the Lewis acid sites of ca-HAp, representing the first experimental evidence reported in the literature. Additionally, the P 2p spectra in Figures 4b and 4h also present new observable species with a shift of 1.6 eV (with respect to the original states at 132.4 and 133.2 eV of P 2p_3/2_ and P 2p_1/2_, respectively), which we attribute to the presence of *N_2_ in the Brønsted acid sites of the aforementioned ca-HAp. Moreover, the Ca^2+^ sites can be considered as 6-fold coordinated with oxygen atoms, and thus, the presence of adsorbed *N_2_ in such acid sites might also contribute to the shift observed in P 2p. Although O 1s is normally fitted considering a single P–O oxidation peak at ∼530.6 eV due to the resonance structure of the PO_4_^3–^ tetrahedra,^60^ such resonance might be no longer maintained because of the presence of *N_2_, resulting in the differentiation of single- and double-bonded oxygens. Accordingly, the O 1s spectra in Figure 4c have been fitted using two peaks at 532.9 eV (P=O) and 530.7 eV (P–O). Based on the elemental composition of these compounds, the ratio between the O 1s areas has been set to 1:4, in excellent agreement with the experimental data. The same ratio constraint has been considered for other measuring conditions (i.e., without UV irradiation), resulting in a proper fitting.

**Figure 4:**
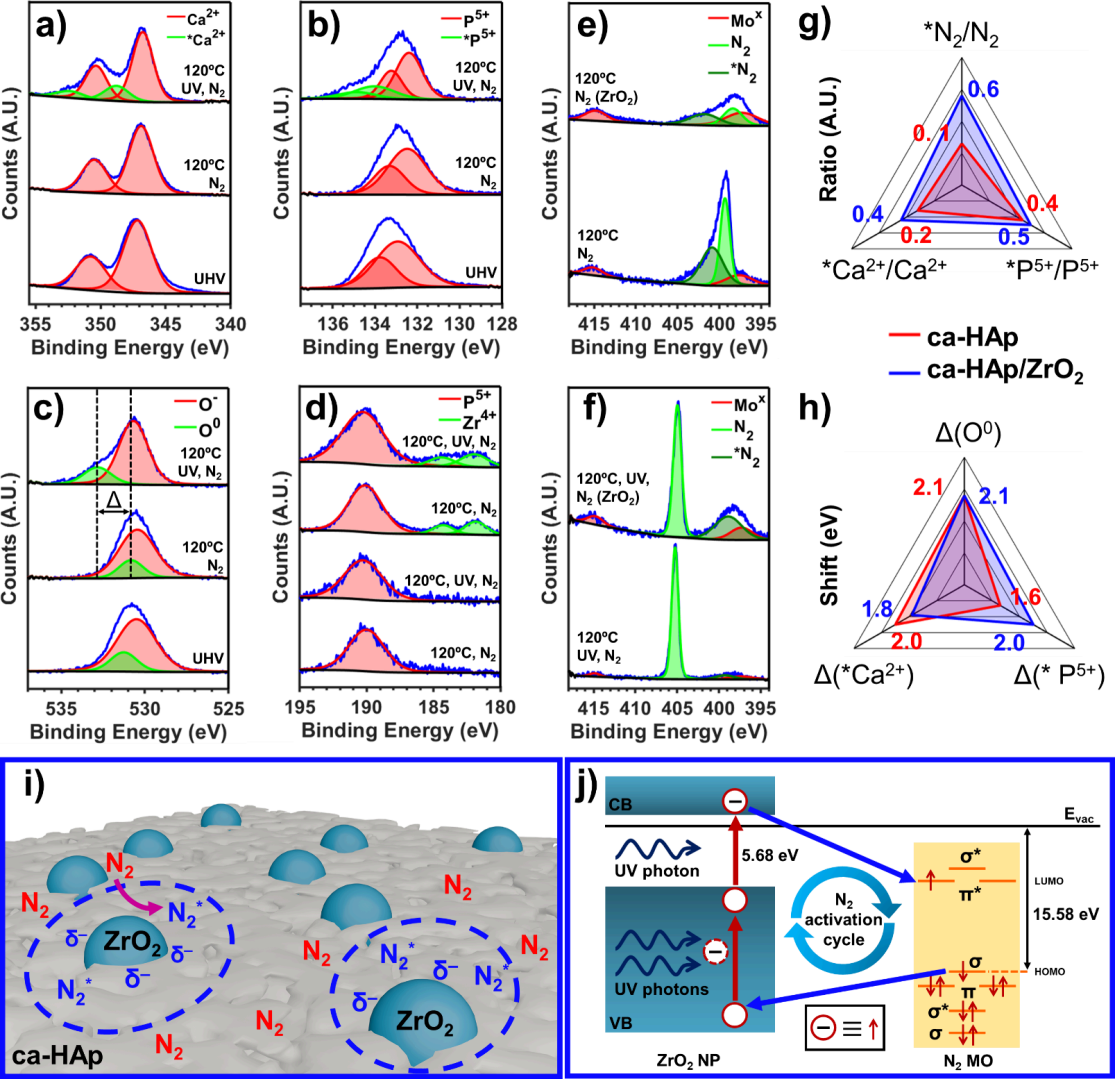
NAP-XPS measurements under different *in stiu* conditions (UHV: ultrahigh vacuum, N_2_ pressure: 1.0 mbar, 120 °C, UV irradiation through a UPS source). (a–c) Ca 2p, P 2p, and O 1s high-resolution regions of the ca-HAp catalyst, respectively. (d) P 2s/r 3d high-resolution region of ca-HAp (i.e., no ZrO_2_) and ca-HAp/ZrO_2_ nanocomposite. (e) N 1s/Mo 3p high-resolution region comparing the ca-HAp and ca-HAp/ZrO_2_ catalysts measured at 120 °C in a N_2_ atmosphere but without UV irradiation. (f) N 1s/Mo 3p high-resolution region comparing ca-HAp and ca-HAp/ZrO_2_ catalysts measured at 120 °C, in a N_2_ atmosphere, and under UV irradiation. (g, h) Ratio and shifts of the *Ca^2+^, *P^5+^, and O^0^ species attributed to the presence of adsorbed dinitrogen (*N_2_). (i) Scheme of the proposed mechanism including the initial activation of N_2_ promoted by the ZrO_2_ NPs and the subsequent migration/adsorption to the ca-HAp binding sites. (j) π-Backdonation mechanism sketch.

On the other hand, the role of ZrO_2_ NPs in ca-HAp/ZrO_2_ was also studied in Figure 4d. For completeness purposes, ca-HAp analyses in the same region and conditions are also shown (first two bottom spectra). Surprisingly, neither any shift nor new species appear in the Zr 3d region (181.8 and 184.3 eV for Zr 3d_5/2_ and Zr 3d_3/2_, respectively), discounting any stable adsorption of N_2_. Therefore, the results suggest that ZrO_2_ NPs do not provide any binding sites and are only responsible for attracting and photoactivating the N_2_, which quickly migrates and binds to ca-HAp (Figure 4i). Although we studied this behavior for a similar system (i.e., zyrconyl chloride) through DFT calculations,^42^ this is the first time that this effect has been demonstrated experimentally. Additionally, this result highlights the importance of the interaction between ca-HAp and ZrO_2_ NPs achieved through the TSP treatment drastically reducing the electronic transport losses at their interface.^49,61^

The N 1s region was examined for ca-HAp and ca-HAp/ZrO_2_ without UV irradiation (Figure 4e) and under UV irradiation (Figure 4f). As can be seen in both figures, while N_2_ gas (405.0 eV) can be observed at the surface of both samples, a non-negligible amount of *N_2_ (398.9 eV) is detected for ca-HAp/ZrO_2_, further validating our previous conclusions that N_2_ is molecularly adsorbed on the surface. Interestingly, a significant binding energy shift of the molecularly adsorbed N_2_ on the surface of the catalyst can also be observed, highlighting changes in the electronic properties (i.e., surface charge accumulation, among others) of the surface of the catalyst occurring after the TSP treatment. Unfortunately, small traces of molybdenum (Mo) were measured and subsequently identified as a contaminant during the HAp pore generation procedure. Even though Mo was found in both samples, discarding unknown synergies (i.e., ca-HAp yield remains low), exhaustive controls were performed to discard its catalytic contribution, consisting of (1) analysis of the Mo oxidation state, revealing the presence of the noncatalytically active MoO_2_ phase (Figure S16), (2) preparation and XPS analyses of a Mo-free catalyst (Figure S17), and (3) N_2_-to-NH_3_ reactions with Mo-free catalyst, reaching the same yields (Figure S18). Details and discussion of these results are provided in the Supporting Information (SI).

The NAP-XPS measurements allow elucidating the mechanism of the reaction by comparing the evolution of *N_2_ on the active binding sites (referred to as *). To do so, the ratios and shifts of *N_2_/N_2_, *Ca^2+^/Ca^2+^, and *P^5+^/P^5+^ of ca-HAp and ca-HAp/ZrO_2_ are displayed in Figures 4g and 4h, respectively. It is evident that the photoactivation of N_2_ through the ZrO_2_ π-backdonation mechanism (Figure 4j) is crucial, increasing the *N_2_/N_2_ ratio from 0.1 for ca-HAp to 0.6 for ca-HAp/ZrO_2_. Moreover, the Lewis acid sites (Ca^2+^) appear to be more active than the irreversible Brønsted acid sites (P-OH). Accordingly, the analysis of the NH_3_ adsorbed on the ca-HAp/ZrO_2_ after the reaction was carried out (Figure S19), revealing a negligible amount of NH_3_ adsorbed, in complete agreement with the NAP-XPS results. Analysis of the binding energy shifts shows that *Ca^2+^ remains stable while increasing the amount of adsorbed *N_2_ (i.e., almost no shift is observed), while the binding energy shift of *P^5+^ increases from 1.6 to 2.0 eV. Apart from the expected shift due to *N_2_ in the Brønsted acid sites, this phenomenon could be attributed to the increasing contribution of the oxygen-coordination with the *Ca^2+^sites. Indeed, the shift observed in *P^5+^ is around ∼4.8 times that observed for oxygen. For completeness, a similar NAP-XPS analysis has been carried out comparing different temperature reactions (*T* = 25, 95, and 120 °C). Results with their corresponding spectra are presented in Figures S20–S24. Overall, a clear synergy between photoactivation (ZrO_2_) and high activity of Ca^2+^ binding sites (ca-HAp) is demonstrated experimentally. Finally, it is worth highlighting that with the aim to further approach real working conditions, we have also performed NAP-XPS measurements introducing N_2_ and water vapor in the chamber. As presented in Figure S25, the enhanced *N_2_ for ca-HAp/ZrO_2_ is further corroborated, observing also differences in the O 1s region corresponding to water species (see the discussion in the figure caption). The specific quantification of the species mentioned above is presented in Figure S26.

### DFT Calculations of ca-HAp and ZrO_2_

Although the importance of Ca^2+^ as binding sites has been highlighted by the NAP-XPS studies, there exist two crystallographic nonequivalent Ca^2+^ cations in the ca-HAp lattice. In terms of the 6-fold oxygen coordination, Ca­(I)^2+^ forms a distorted octahedron including one hydroxyl group, while Ca­(II)^2+^ forms a metaprism arising from six different PO_4_^3–^ tetrahedra (see Figure 1a). Therefore, DFT calculations have been carried out to determine the most probable active crystallographic Ca^2+^ site by means of computing the adsorption energies depending on the reaction temperature (smearing value has been adjusted at 298 and 393 K) and the exposed crystallographic plane (according to the HRTEM results derived from Figure 2).

Such DFT calculations are not trivial, as the standard periodic boundary conditions of the unit cell achieve extensive dimensions due to the presence of vacancies and oriented OH^–^ groups generating asymmetries. Therefore, a supercell consisting of 168 atoms has been considered. All the details can be found in the Methods section. Because of the high density and proximity of Ca^2+^ binding sites, the adsorption energies have been computed considering different adsorption mechanisms such as adsorption in single Ca^2+^ binding sites or dual sites (Ca­(I)–Ca­(I), Ca­(II)–Ca­(II), or Ca­(I)–Ca­(II)), among other configurations, thus achieving complete binding site exploration.

The most relevant DFT results obtained for ca-HAp are presented as a heat map in Figure 5. Although the N_2_ adsorption on Ca­(II) sites cannot be discarded, the single N_2_ adsorption on single Ca­(I) (HAp-2 case) shows the highest adsorption energy. Moreover, the (001) plane, which is measured due to the existence of the TSP superstructure (see Figure 2) is postulated as the most active crystallographic plane. Certainly, the Ca­(I) coordination with the OH^–^ group and by extension the enhanced electronic pathway sketched in Figure 1b explain the fast evolution of intermediates toward NH_3_, which propitiates the fast desorption of the product. At the same time, the effectiveness of the vacancy generation and electron transfer strategies achieved by the TSP treatment are also corroborated. On the other hand, no significant changes can be observed when increasing the temperature, which is in agreement with the NAP-XPS results reported in Figure S17, further supporting the stability of the Ca­(I) principal binding sites of the catalyst.

**Figure 5:**
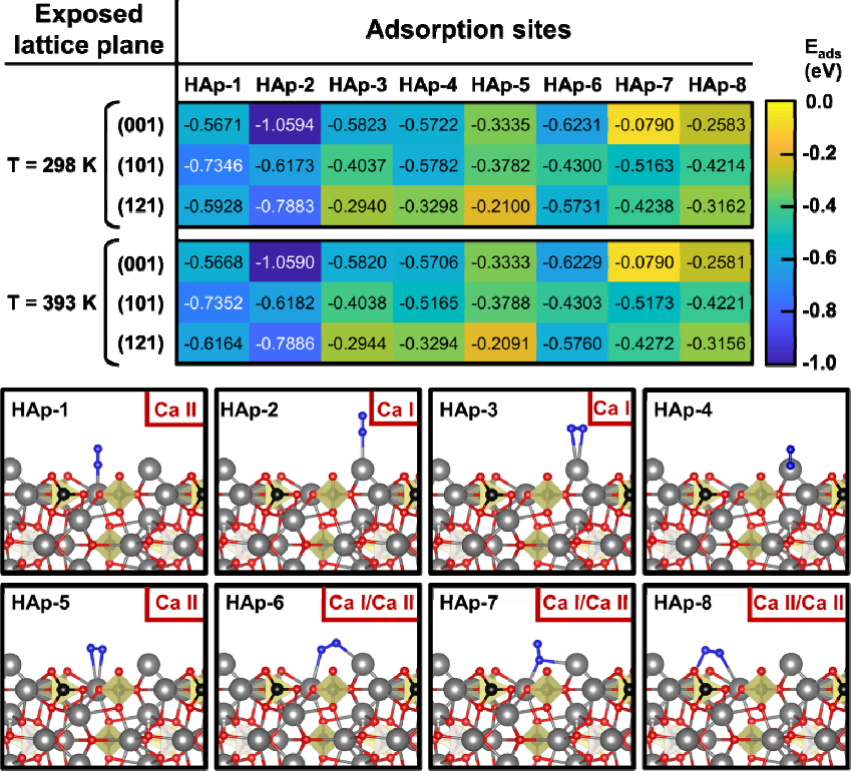
N_2_ adsorption studies performed on ca-HAp preferential exposed planes at 298 and 393 K by means of DFT calculations.

Due to the role of water acting as a proton source, competitive adsorption in the calcium acid sites is expected. In this sense, the multiple binding sites observed in Figure 5 ensure simultaneous adsorption of N_2_ and H_2_O. Nonetheless, DFT adsorption studies considering the most probable binding site (HAp-2) and a water molecule relaxed in 4 different positions nearby have been carried out, as shown in Figure S27. Results, listed in Table S1, further support the prior adsorption studies, as negative adsorption energies are obtained, even considering the energy interaction between the relaxed N_2_ and H_2_O molecules. Overall, despite the competitive nature of the reaction, DFT calculations support the viability of the mechanisms proposed for the reaction.

Similarly, DFT adsorption studies were performed on ZrO_2_. Unlike ca-HAp, the results depicted in Figure 6 show clear preferential adsorption sites consisting of N_2_ anchoring with four Zr^4+^ atoms (Z-3 case). Interestingly, this is the only configuration showing higher adsorption energy than the ca-HAp sites, which in general presents a higher density of potential adsorption sites. Because of the singular characteristics of the Z-3 case (i.e., strong positively charged environment for both nitrogen atoms), a change in the N_2_ adsorption configuration is expected after its activation through the π-backdonation mechanism. According to the heat maps derived from both DFT calculations (Figures 5 and 6), a nitrogen spillover toward the Ca­(I) sites of ca-HAp is more probable considering both the adsorption energies and the specific environment of Ca­(I) discussed above, which promote the subsequent nitrogen reduction reaction. In this context, it is worth highlighting the adequate structural union achieved at the ca-HAp and ZrO_2_ NP junction, which enables charge transfer and atom migration. In this sense, the water content, acting as the proton supply, might also play a crucial role by closing the overall electronic pathway, as no external electrical potentials are applied. The complete role of water is currently being investigated. These conclusions are supported by the NAP-XPS results presented in Figure 4d, which discard a stable adsorption of N_2_ onto the ZrO_2_ NPs, as no change in the binding energies is measured.

**Figure 6:**
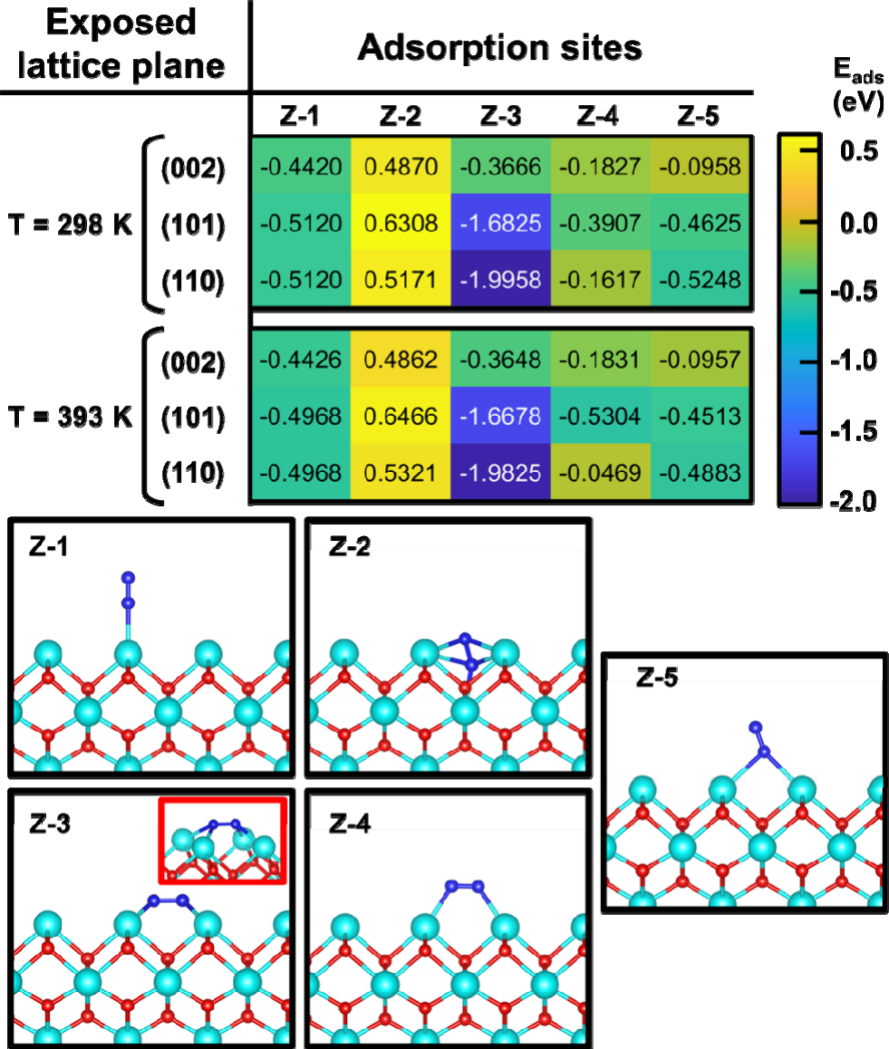
N_2_ adsorption studies performed on tetragonal ZrO_2_ preferential exposed planes at 298 and 393 K by means of DFT calculations.

### DFT Calculations of ca-HAp/ZrO_2_ as a Nanocomposite

A representative ca-HAp/ZrO_2_ model has been built to obtain further insights into the catalytic mechanism proposed. To do so, a ZrO_2_ NP consisting of 42 atoms (see Figures 7a and 7b) has been used. All of the computational details are provided in the Methods section. It is worth mentioning that the ZrO_2_ NPs in the nanocomposite are much bigger than the one in the model (as would be computationally unfeasible). In this sense, one of the most important aspects of the reaction mechanism is the nitrogen spillover; thus, we assume that the model used is valid and representative, as the phenomena under study occur at the interphase between ca-HAp and ZrO_2_ NPs. Nonetheless, when reducing the size of ZrO_2_ NPs, it is important to consider different ca-HAp and ZrO_2_ interfacial interactions, as the ZrO_2_ NPs can be immobilized in different nonequivalent crystallographic positions with respect to the ca-HAp crystal lattice: (1) northeast, (2) southeast, and (3) centered (just on top of the OH^–^ columns). Therefore, we have carried out nudged elastic band (NEB) calculations to determine the thermodynamic feasibility and associated energetic barriers of the nitrogen spillover.

**Figure 7:**
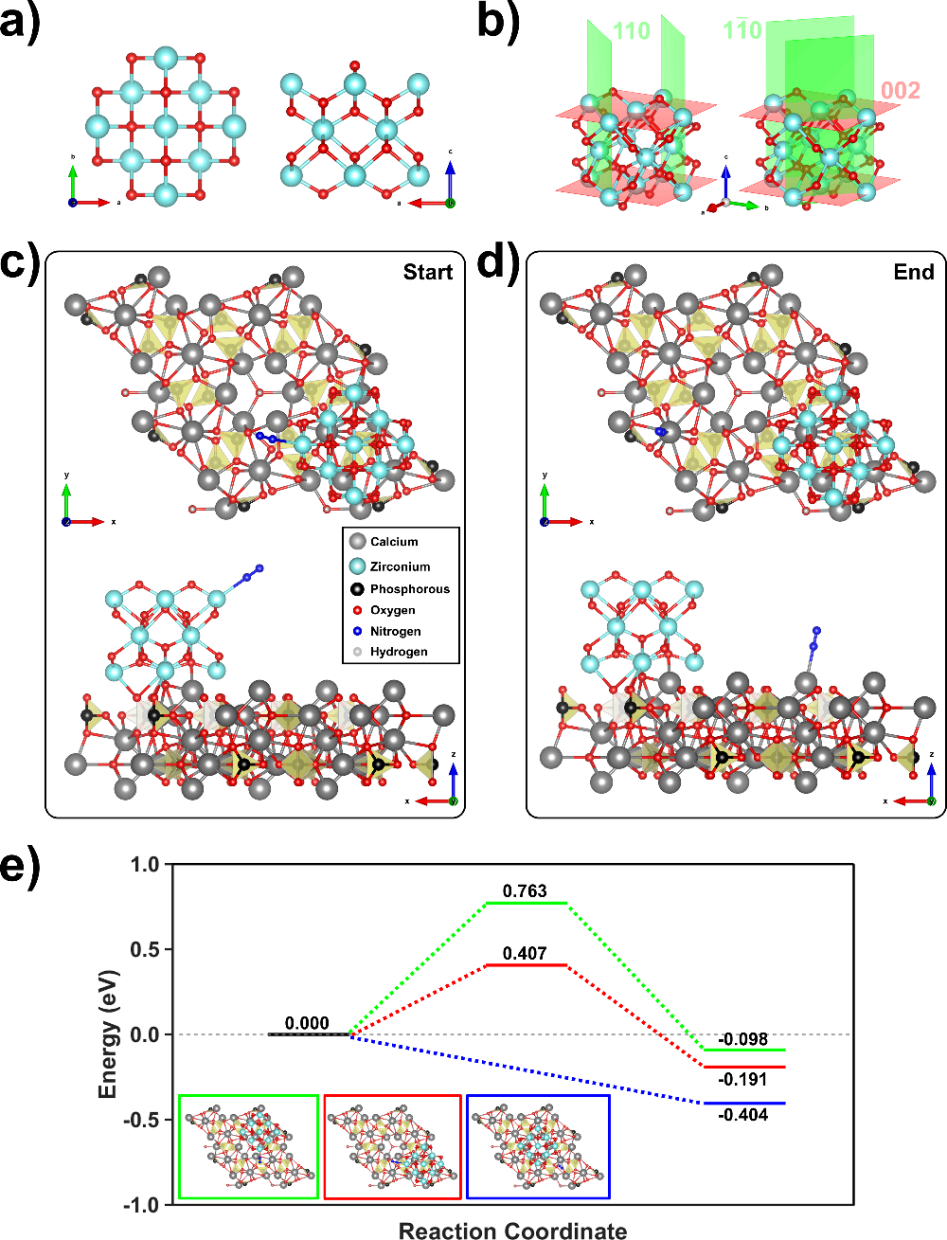
(a, b) Model of the ZrO_2_ NP used for the simulations. (c, d) Starting and ending points used to perform the NEB calculations related to the nitrogen spillover mechanism proposed. The ZrO_2_ NP has been located at the southeast crystallographic position with respect the ca-HAp lattice. (e) Energy barriers associated with the nitrogen spillover. In the insets of the figure are depicted the three considered cases under study. Accordingly, depending on the crystallographic position of the ZrO_2_ NP, the final energetic balance with their associated barrier varies.

According to the previous results, NEB calculations were performed considering a relaxed nitrogen molecule close to Zr^4+^ atoms (starting point), while the final point was determined by relaxing the nitrogen molecule close to the Ca­(I) sites of ca-HAp. Figures 7c and 7d present the starting and final points for the position (2) southeast, while in Figures S28 and S29 are depicted the starting and final points for cases (1) northeast and (3) centered. Furthermore, in the inset of Figure 7e all three cases are schematically represented: (1) green, (2) red, and (3) blue. As can be seen in Figure 7e, a favorable energetic transition is obtained for all the cases studied, supporting the proposed spillover mechanism. Interestingly, the centered position (3) does not show any associated energetic barrier, which has been attributed to the fact that the nitrogen molecule is not strongly adsorbed to the Zr^4+^ because of steric interactions between ca-HAp and ZrO_2_. Herein, it is worth highlighting that all three interfacial interactions are expected due to the real size of the ZrO_2_ NPs. Nevertheless, and assuming nitrogen activation through the π-backdonation mechanism, the red approach interfacial interaction appears to be the most plausible considering the values obtained for the energetic barriers.

On the other hand, the elucidation of the reaction pathway has been assessed by calculating the differences in adsorption energies associated with the reaction coordinates of the N_2_-to-NH_3_ reaction pathway proposed in the literature.^11,38,42^ Results are summarized in Figure 8. Differences are referenced to the most probable adsorption site, HAp-2 (see Figure 5). As it can be seen, most of the reaction pathways studied are energetically favorable, demonstrating the viability of the process. It is worth highlighting also that most of the energies of the reaction coordinates homogeneously decrease, leading to almost direct cascade reactions, which are in complete agreement with the extremely high selectivity toward NH_3_ and lack of intermediates measured experimentally. Furthermore, the reaction pathway presents 4 potential NH_3_ desorption steps. This computational result further supports the experimental observations, as it explains the high NH_3_ desorption capacity of the catalytic system reported (i.e., NH_3_ is mainly found in the supernatant) while establishing, for the first time, a ca-HAp complete mechanism for nitrogen fixation into ammonia.

**Figure 8:**
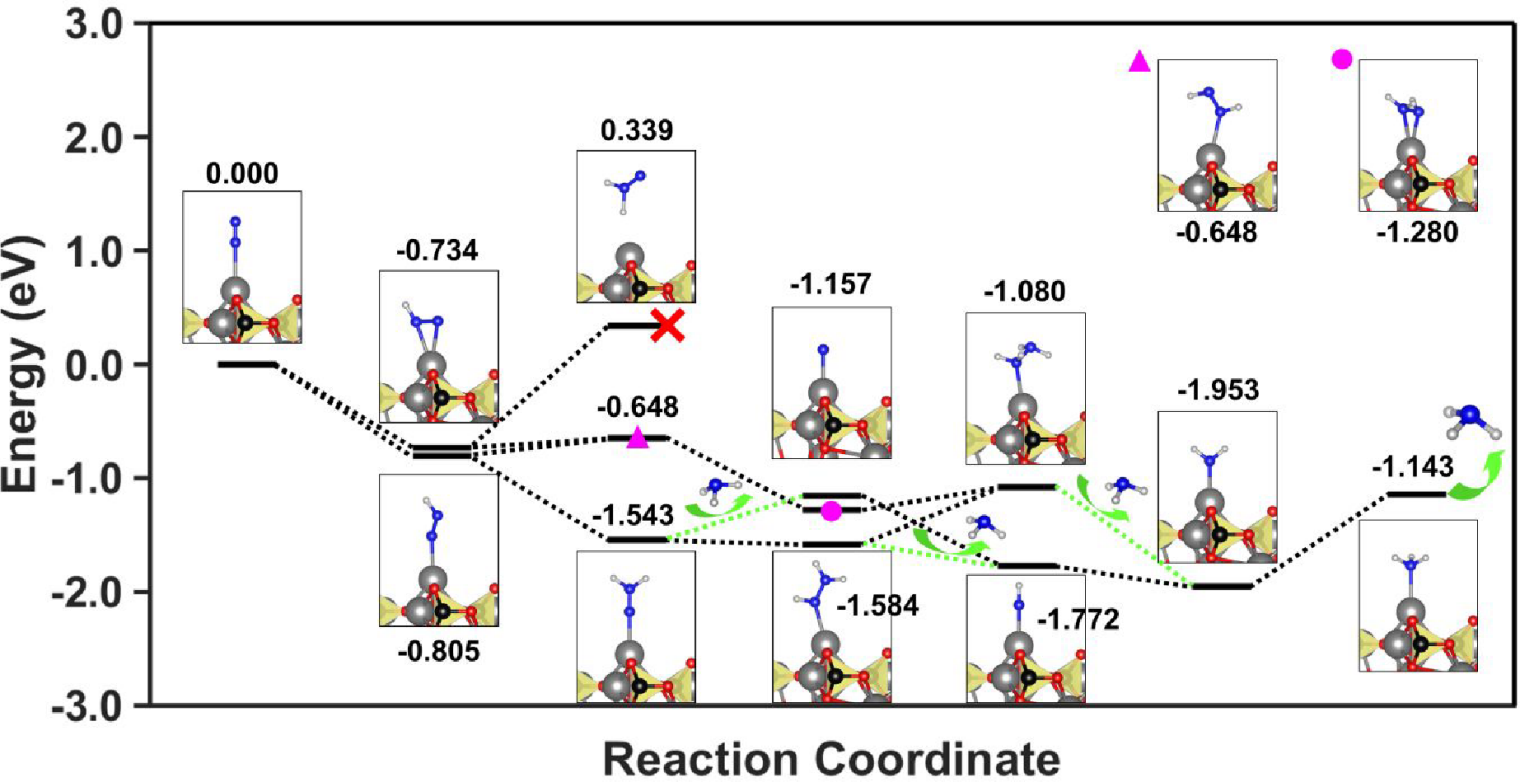
Reaction pathway calculated for a (001) ca-HAp slab.

Finally, it is worth mentioning again the role played by water in the reaction mechanism. Based on previous works reported for ca-HAp, a water-splitting reaction is assumed.^43–45,49^ The energy barrier associated with the O–H cleavage and proton incorporation in the second reaction coordinate of Figure 8 have been assessed through NEB calculations in Figure S30. Accordingly, an energetic barrier of 3.713 eV is obtained for the transition state. Although such a value could be overcome by different sources such as temperature,^44,45^ the Tauc plot presented in Figure S31 (derived from the UV–vis spectrum of Figure S14) reveals a first band gap transition at 4.09 eV. Therefore, the feasibility of the whole mechanism including the role of water as proton supply is demonstrated.

## Conclusions

ca-HAp/ZrO_2_ nanocomposites have been successfully designed and synthesized to efficiently carry out N_2_-to-NH_3_ reactions under mild conditions (120 °C, 6 bar of N_2_, and 20 mL of H_2_O) and low-power UV light irradiation. More specifically, an ammonia production of ∼1.6 ± 0.2 mmol_NH3_·g_c_ is obtained when decorating ca-HAp with ZrO_2_ NPs. Certainly, a clear synergy arises from such nanocomposites, as the ammonia yield obtained separately (i.e., ca-HAp and/or ZrO_2_ alone) is, at least, 1 order of magnitude smaller. More specifically, the combination of *in situ* experimental evidence and DFT results has allowed elucidating the mechanism for the N_2_-to-NH_3_ reaction catalyzed by ca-HAp/ZrO_2_ nanocomposites. Hence, the results confirm the advantageous synergy between the π-backdonation mechanism occurring as a first step in the ZrO_2_ NPs, followed by the fast migration of the activated *N_2_ species to the binding sites of ca-HAp, resulting in their posterior reduction to NH_3_ due to the enhanced binding site density and electronic properties. It is worth stressing again that ca-HAp acts not only as a mechanical support for ZrO_2_ NPs but also as an active element catalyzing the reaction. This study paves the way to the design and development of novel green catalysts based on such synergistic approaches capable of promoting a viable transition toward the green N_2_-to-NH_3_ reactions, as demonstrated by the green ca-HAp/ZrO_2_ nanocomposite presented.

## Methods

### Reagents

Ammonium phosphate dibasic [(NH_4_)_2_HPO_4_; purity ≥ 99.0%], ammonium hydroxide solution 30% (NH_4_OH; purity: 28–30%), calcium nitrate tetrahydrate Ca­(NO_3_)_2_·4H_2_O (ACS reagent, 99–103%), and Pluronic F-127 were obtained from Sigma-Aldrich. Commercial ZrO_2_ nanoparticles (TZ-3YSB-C; yttria-stabilized ZrO_2_ showing enhanced thermal stability) were purchased from TOSOH Corp. Ethanol (CH_3_CH_2_OH, purity ≥ 99.8%) was purchased from Honeywell. Deionized water was obtained using a Milli-Q water purification system from MILLIPORE S.A.

### Preparation of ca-HAp/ZrO_2_ Catalysts

Hydroxyapatite synthetic powder was prepared by adding 15 mL of 0.5 M (NH_4_)_2_HPO_4_ deionized water solution to 25 mL of 0.5 M Ca­(NO_3_)_2_/ethanol (adjusted to pH 11 with NH_4_OH), keeping a Ca/P stoichiometric ratio of 1.66. Precipitated powder was refined by applying a hydrothermal process, 150 °C for 24 h, using a Digestec DAB-2 autoclave. Samples were centrifuged and then lyophilized for 3 days to obtain a white HAp powder. Pluronic hydrogel was obtained by mixing with a FlackTek SpeedMixer at 3500 rpm for 5 min 25 g of deionized water with 25 g of Pluronic F-127 and adding subsequently 50 g of Pluronic polymer.^44^

Porous HAp cubes were obtained by (1) mixing the as-prepared HAp powder with the liquid Pluronic hydrogel following a 40/60% w/w ratio at 5 °C, obtaining an ink, which was later shaped into cubes, and (2) calcinating the HAp/Pluronic cubes at 1000 °C, eliminating the hydrogel from the HAp scaffold. ZrO_2_ NP deposition onto the calcined HAp porous scaffolds was carried out by means of electrophoretic deposition (EPD), using 0.5 mM ZrO_2_ in 10/90% v/v water/ethanol suspensions (optimized conditions, Figures S7 and S8). The HAp porous scaffold was placed covering the positive electrode and then immersed inside the suspension media followed by the application of a 6 V (with a Multi Autolab/M101 from Metrohm) difference between both stainless-steel electrodes, thus making possible the deposition process. The scaffolds were extracted after the EPD and calcinated at 1000 °C, promoting the sintering between HAp and ZrO_2_ NPs, thus obtaining HAp/ZrO_2_ samples.

The catalytic activation of the hydroxyapatite in the HAp and HAp/ZrO_2_ porous scaffolds was achieved through the thermally stimulated polarization process, consisting of applying a DC voltage of 500 V (through two stainless steel AISI 304 electrodes separated 4 cm) at 1000 °C for 1 h. After that time, samples were cooled while maintaining the voltage for 30 min, finally obtaining ca-HAp and ca-HAp/ZrO_2_.

### Characterization of the Catalysts

High-resolution electron microscopy (HRTEM) images were acquired by using a JEOL 2100 microscope equipped with an LaB6 thermionic electron gun (acceleration voltage of 200 kV). Samples were dispersed in deionized water, sonicated, and drop-casted on a grid with holey-carbon film. Morphology studies were performed using a Zeiss Neon40 scanning electron microscope (SEM) equipped with a GEMINI column with a Schottky field emitter. Secondary and backscattered electron detectors were used for acquiring sample surface images. Both pore and NP size distributions were calculated from SEM images using 100 NPs as representative sample data to ensure the values’ precision. Brunauer–Emmett–Teller (BET) surface area analyses were carried out with a Micrometrics ASAP 2000 system using N_2_ and with sample degasification conditions of 1 h at 90 °C followed by 4 h at 300 °C to ensure complete gas desorption from the samples. Wide-angle X-ray scattering (WAXS) spectra were acquired using a Bruker D8 Advance model with a Bragg–Brentano 2θ configuration and Cu K_α_ radiation (λ = 0.1542 nm). Measurements were performed in a 2θ range of 20–60° in steps of 0.02° and a scan speed of 2 s, using a one-dimensional Lynx Eye detector.

Ammonia detection was performed by means of ^1^H NMR spectroscopy. All spectra were acquired by using a Bruker Ascend 400 spectrometer operating at 400.1 MHz. The chemical shift was calibrated using tetramethylsilane (TMS) as internal standard, and liquid supernatant/dissolved catalysts were mixed with 100 μL of DMSO-*d*_6_. Sample acquisitions consisting of 512 scans were recorded in all cases.

Raman spectroscopy analyses were performed using an inVia Qontor confocal Raman microscope (Renishaw) equipped with a Renishaw Centrus 2957T2 detector and a 532 nm laser. Sample mappings consisting of 32 single-point spectra were averaged to obtain representative data. Zeta (ζ)-potential measurements for EPD optimization studies were performed with a NanoBrook 90Plus Zeta equipped with an AQ-1321. UV–vis spectra were acquired using a Shimadzu UV-3600 equipment with an ISR-3100 integrating sphere accessory (inner diameter 60 mm, PMT and PbS detectors).

### N_2_-to-NH_3_ Fixation Reactions

Nitrogen fixation reactions were performed in a batch reactor consisting of a reaction chamber coated with a perfluorinated polymer (120 mL). The specific sketch of the reactor is presented in Figure S11. Gas charge and extraction were achieved through inlet and outlet valves, respectively, equipped on the reactor. A quartz tube placed in the center of the reactor (also protected with perfluorinated polymer) allowed direct sample irradiation by using UV light (GPH265T5L/4, 253.7 nm). All reactions were carried out at 120 °C and 6 bar of N_2_, with 20 mL of deionized water for 72 h. Catalysts were placed at a medium height inside the chamber, thus ensuring they were not immersed in the water. After the reaction, supernatant products and catalyst-adsorbed products were analyzed. Protonation of the ammonia into ammonium^41^ was achieved by adding 100 μL of 0.2 M H_2_SO_4_ solution to 1 mL of supernatant, whereas for the catalyst-adsorbed products, catalyst dissolution and ammonia protonation were performed as a single-step process by placing the catalyst into 1 mL of 7.6 mM H_2_SO_4_ solution and sonicating. Yield quantification was performed by ^1^H NMR area integration comparing to a reference standard (NH_3_ Panreac, purity > 99.8%).

### NAP-XPS Studies

*In situ* near-ambient-pressure XPS experiments were conducted in an ultra-high-vacuum system (Specs GmbH) equipped with a Phoibos NAP-150 hemispherical analyzer and a monochromatic Al Kα source (*h*ν = 1486.7 eV). N_2_ pure gas and N_2_ with H_2_O vapor (at a 1:1 ratio) were leaked into the sample chamber using a mass flow controller. Pressure was held constant during measurement by means of a throttle valve regulating the pumping cross section of a differential pumping stage. A diaphragm capacitance gauge was used to measure the pressure, which was 1.0 mbar during the measurement. Sample heating was performed by means of a near-infrared laser illuminating the backside of the sample. A NAP-UPS source (Specs GmbH) operating with He gas was used as the source of UV irradiation (*h*ν = He I radiation, 21.2 eV).

Survey spectra as well as high-resolution Ca 2p, P 2p, O 1s, N 1s, Mo 3d, and C 1s spectra were recorded while the sample temperature was 25, 95, and 120 °C, respectively. The pass energy for the survey spectra was 100 eV, while the high-resolution spectra were recorded with a pass energy of 20 eV. For analysis, all spectra were calibrated using the carbon 1s peak at 284.5 eV. Peak data fitting was performed using Tougaard, U Poly Tougaard, and Shirley background types for the Ca 2p, N 1s, and the remaining elements, respectively. LA (1.643) line shape was used.

### DFT Simulations

DFT calculations were performed using the Quantum ESPRESSO (QE) v.7.2 package^62^ to calculate nitrogen adsorption energies onto HAp and ZrO_2_. A generalized gradient approximation (GGA) with the Perdew–Burke–Ernzerhoff (PBE) formalism was used for exchange–correlation energy treatment.^63^ Ultrasoft pseudopotentials (PP) were acquired from the standard solid-state pseudopotentials (SSSP Precision v 1.3.0) library,^64^ and a Hubbard correction for Zr 4d electronic orbitals with *U* = 8.0 eV was performed for achieving a better treatment of them.^65^ The kinetic energy cutoff for wave functions was set to 30 Ry, while for charge density it was set to 300 Ry. Reaction temperatures were simulated by applying a Fermi–Dirac function smearing scheme with broadening consisting of 1.9 × 10^–3^ Ry (298 K) and 2.5 × 10^–3^ Ry (393 K).

ca-HAp slab models were designed according to hexagonal HAp (space group symmetry *P*6_3_/*m*) with experimental cell parameters *a* = *b* = 9.432 and *c* = 6.881 Å. Hydroxyl group vacancies were generated in the lattice columns equivalent to 50%, as seen elsewhere,^46^ and the total charge for the system was corrected according to the number of missing charges. The polarization effect was modeled by reorienting the hydroxyl groups toward a specific direction and relaxing their positions along the *c*-axis to minimize steric hindrances. According to HRTEM, three slab supercells 2 × 1 × 2, 2 × 1 × 2, and 2 × 2 × 1 for crystallographic planes (001), (101), and (121), respectively, were assembled (168 atoms each) with 30 Å vacuum thickness. Gamma point calculation was used to reduce computational time due to the slab size. Tetragonal ZrO_2_ slab models (space group symmetry *P*4_2_/*nmc*) were designed with experimental cell parameters *a* = *b* = 3.596 Å and *c* = 5.177 Å. Three slab supercells 3 × 2 × 2, 2 × 2 × 2, and 2 × 2 × 2 for crystallographic planes (002), (101), and (110), respectively, were prepared (96 atoms each) with 30 Å vacuum thickness.

Adsorption energies were calculated using the formula *E*_ads_ = *E*_slab+N2_ – *E*_slab_ – *E*_N2_ where the energies of the sole slab (*E*_slab_) and the nitrogen molecule (*E*_N2_) are subtracted from the energy of the slab with adsorbed nitrogen (*E*_slab+N2_).

For N_2_ spillover simulations, a ZrO_2_ NP consisting of 42 atoms with exposed planes (002), (110), and (1–10) was assembled and placed on top of a ca-HAp slab with exposed plane (001) according to three different sites (total system: 212 atoms). The ZrO_2_ atoms found at the interface between the NP and slab were relaxed following the *z*-axis direction, allowing the structure to keep its geometry while decreasing the system energy. Nitrogen molecules were placed near the atomic environment for both systems (NP and slab) and allowed to relax to their closest local minimum energy position. By avoiding forced molecular relax into a global minimum energy position, a behavior resembling capture of a passing-by molecule was achieved, as in experimental systems. For ca-HAp the final adsorption site was found to be HAp-2 in all cases. Nudged elastic band (NEB) calculations were performed generating 8 images with a path threshold of 0.2 eV/Å by means of Broyden optimization scheme with automatic climbing images.

N_2_ to NH_3_ reaction pathway intermediate product energies were calculated following the expression *E*_ads_ = *E*_slab+Intermediate_ – *E*_slab_ – *E*_Intermediate_. The HAp-2 position was chosen for adsorbing the intermediates, since it presented the higher adsorption energy for the nitrogen molecule. However, molecular relaxation was not constrained to this specific binding site, enabling some intermediates to relax in two or more binding sites. All the adsorption energies were referred with respect to the nitrogen adsorption energy.

## Supplementary Material



## Notes

Further electrochemical and structural characterization, electrophoretic optimization (Figures S1–10), reaction experiments, stability and schemes (Figures S11–S19), NAP-XPS spectra and results for support (Figures S20–S26), computational studies (Figures S27–S30), and Tauc plot (Figure S31) (PDF)
