# Supplementary material for: The Synergistic Effect of Combining Electron Transfer and Photoactivation in Hydroxyapatite/ZrO2 Nanocomposites Promotes Efficient Nitrogen-to-Ammonia Fixation Reaction
Source: ACS Catal. 2025 Jun 12;15(13):11150–62. doi: 10.1021/acscatal.5c02426 (PMC12235675; doi:10.1021/acscatal.5c02426)
Supplement: Supplementary file 1 [file cs5c02426_si_001.pdf]

ELECTRONIC SUPPORTING INFORMATION

The Synergistic Effect of Combining Electron Transfer and Photoactivation in Hydroxyapatite/ZrO_2 Nanocomposites Promotes Efficient Nitrogen-to-Ammonia Fixation Reaction

Marc Arnau,[1,2] Lukas Pielsticker,[3,] Walid Hetaba,[3] Jordi Casanovas,[4] Pau Turon,[5] Carlos Alemán[1,2,6,*] and Jordi Sans,[1,2,*]*

[1] Departament d'Enginyeria Química, EEBE, Universitat Politècnica de Catalunya, C/ Eduard Maristany, 10-14, Ed. I2, 08019, Barcelona, Spain

[2] Barcelona Research Center in Multiscale Science and Engineering, Universitat Politècnica de Catalunya, C/ Eduard Maristany, 10-14, 08019, Barcelona, Spain

[3] Department of Heterogeneous Reactions, Max Planck Institute for Chemical Energy Conversion, Stiftstr. 34-36, 45470, Muelheim an der Ruhr, Germany

[4] Departament de Química, Física i Ciències Ambientals i del Sòl. Universitat de Lleida Escola Politècnica Superior, C/Jaume II n°69, Lleida E-25001, Spain

[5] B. Braun Surgical, S.A.U. Carretera de Terrassa 121, 08191 Rubí (Barcelona), Spain

[6] Institute for Bioengineering of Catalonia (IBEC), The Barcelona Institute of Science and Technology, Baldiri Reixac 10-12, 08028 Barcelona Spain

** Correspondence to: lukas.pielsticker@cec.mpg.de, carlos.aleman@upc.edu and jordi.sans.mila@upc.edu*

Table S1. Adsorption energy studies for simultaneous N_2 and H_2O molecules in the binding site HAp-2 (related with Figure S27). DFT calculations were performed considering a supercell slab of 2x2x1 (total of 173 atoms) corresponding to the (001) crystallographic plane.

	E_{co-ads} (eV)	$E_{Ads-H2O\|N2}$ (eV)	$E_{Ads-N2\|H2O}$ (eV)	E_{Inter} (eV)
Site 1	-2.3437	-1.7310	-0.4953	0.1174
Site 2	-2.4500	-1.8253	-0.5342	0.0905
Site 3	-2.4512	-1.8204	-0.5374	0.0934
Site 4	-2.4161	-1.8129	-0.4992	0.1040

Considerations for energy calculations

- *Co-adsorption energy:* considering both molecules as one $E_{co-ads} = E_{slab+N2+H2O} - E_{slab} - E_{H2O} - E_{N2}$
- *Incremental adsorption energy:* similar to the procedure followed for N_2 alone $E_{ads-H2O|N2} = E_{slab+N2+H2O} - E_{slab+N2} - E_{H2O}$ & $E_{ads-N2|H2O} = E_{slab+N2+H2O} - E_{slab+H2O} - E_{N2}$. Note that a change is observed due to the new relaxed conformation.
- *Interaction energy:* $E_{slab+N2+H2O} + E_{slab} - E_{slab+N2} - E_{slab-H2O}$

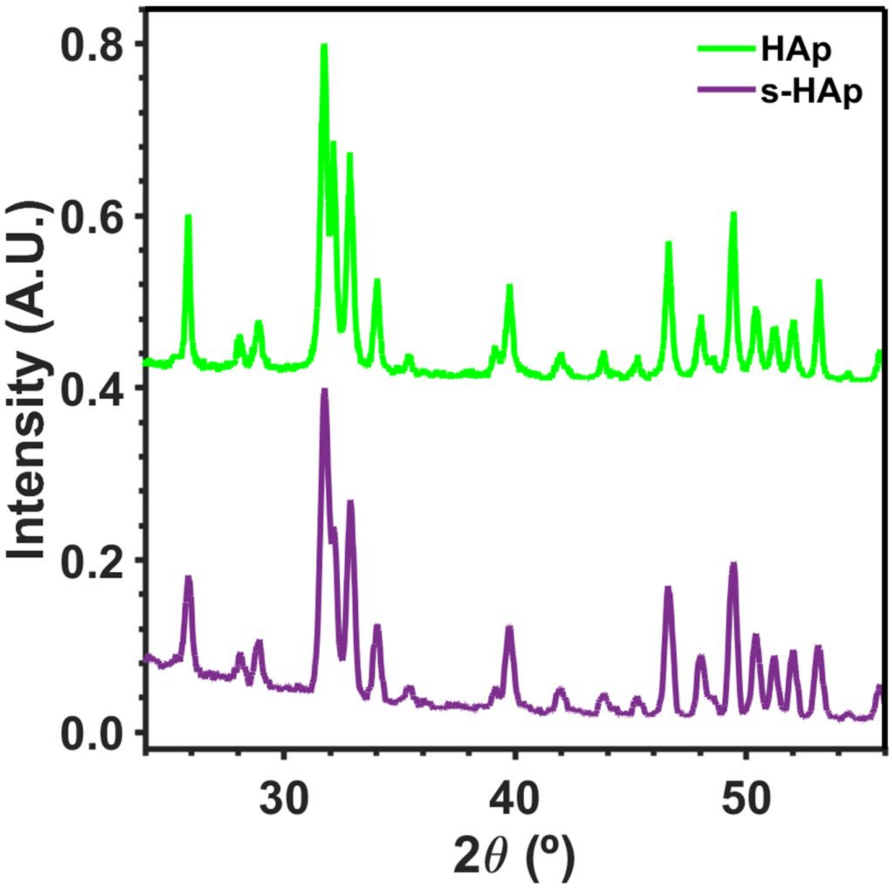

Figure S1. XRD pattern of HAp and s-HAp (*i.e.* HAp after the sintering treatment) samples.

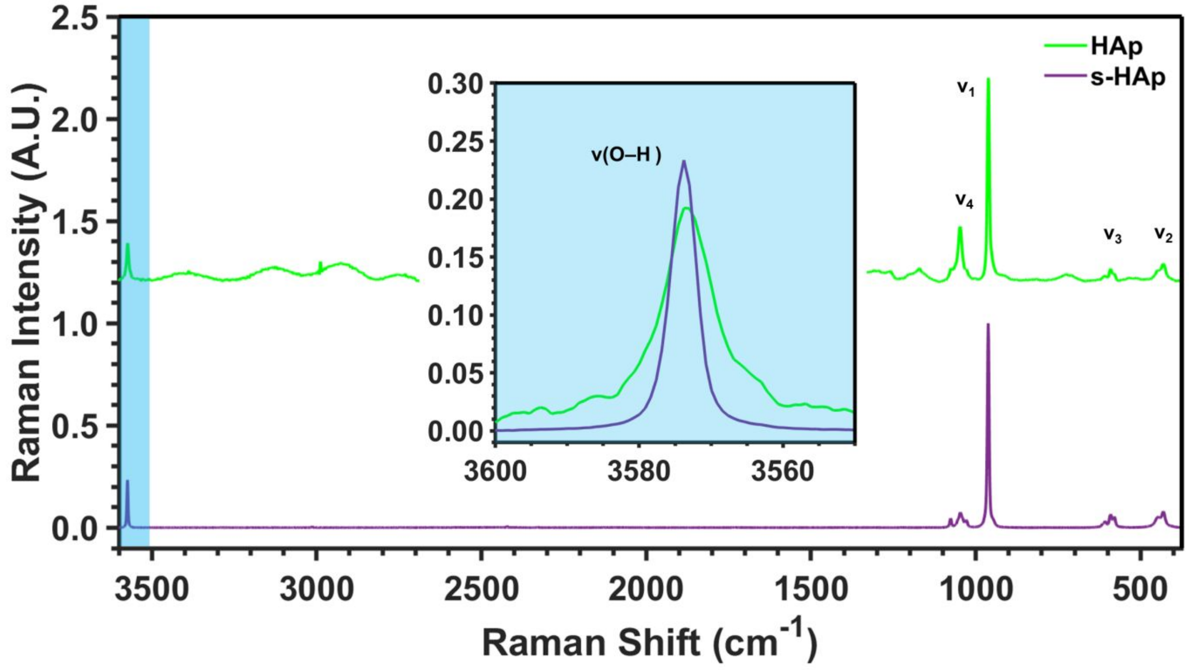

Figure S2. Raman spectra of the HAp and s-HAp (*i.e.* HAp after the sintering treatment) samples. The main vibrations Raman modes of HAp $\nu_{1\text{-}4}$ are highlighted. A clear refinement in the crystalline structure of HAp can be observed by means of the well-resolved and sharper peaks in s-HAp. Moreover, the $\nu_{O\text{–}H}$ characteristic vibration (see inset) evidences the generation of OH^- vacancies in the lattice of s-HAp.

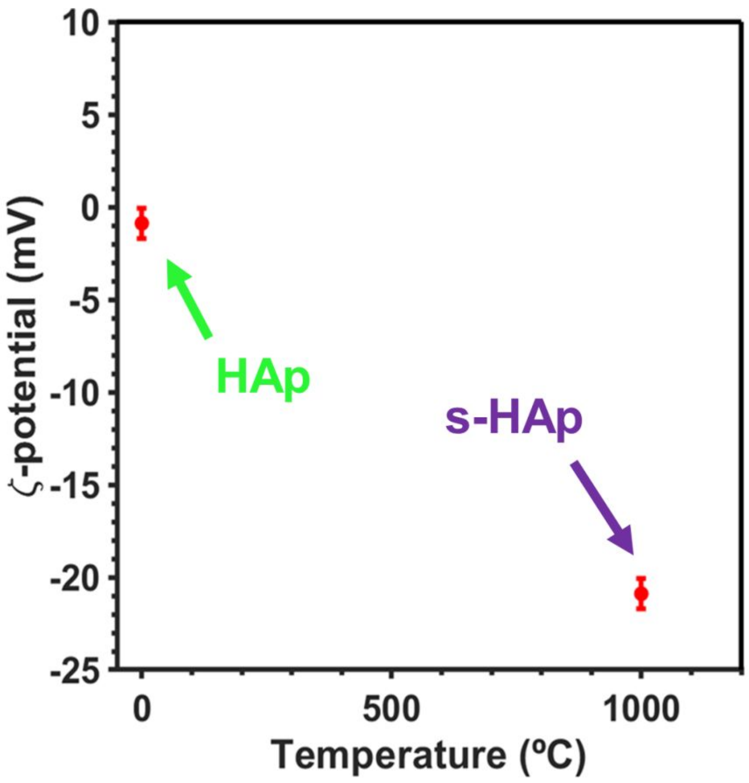

Figure S3. Zeta (ζ)-potential measurements difference between HAp (no sintering temperature, $T_{sintering}$ = 0 ºC) and s-HAp ($T_{sintering}$ = 1000 ºC). The change observed in the superficial charge accumulation is related with the generation of OH^- vacancies reported in Figure S2. The measurements were performed at pH = 7 and in a NanoBrook 90Plus Zeta equipped with an AQ-1321 cell.

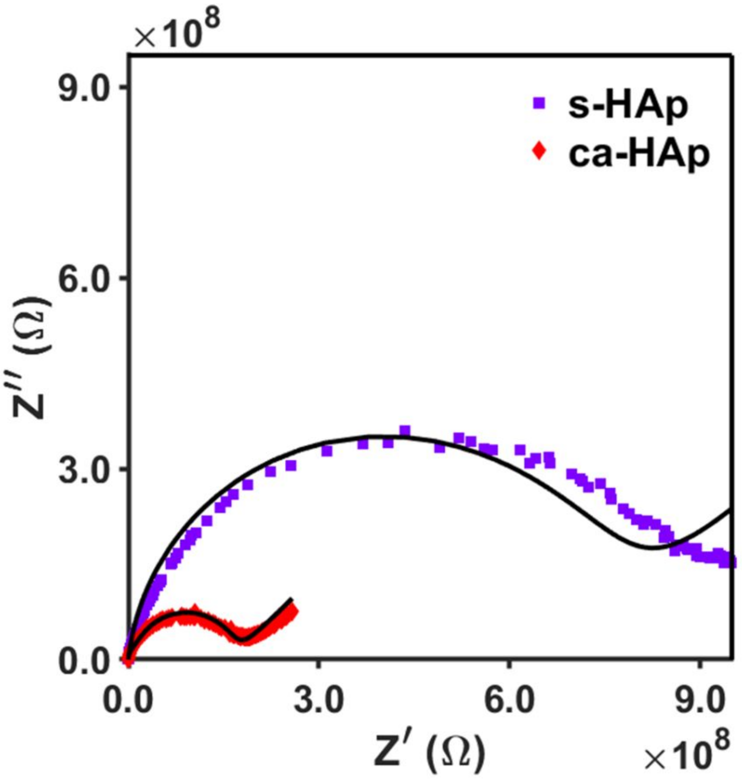

Figure S4. Electrochemical impedance spectra (EIS) for ca-HAp and s-HAp. As it can be observed, towards lower frequencies s-HAp has 50 times more resistive and capacitive behaviour than ca-HAp, revealing the enhanced electrical properties of ca-HAp. Samples were prepared as pellets and analyzed with a solid electrochemical cell consisting of two stainless steel isolated plates hold together by a Teflon case. A 100 mV sinusoidal voltage was applied in a frequency range from 1 MHz to 10 mHz.

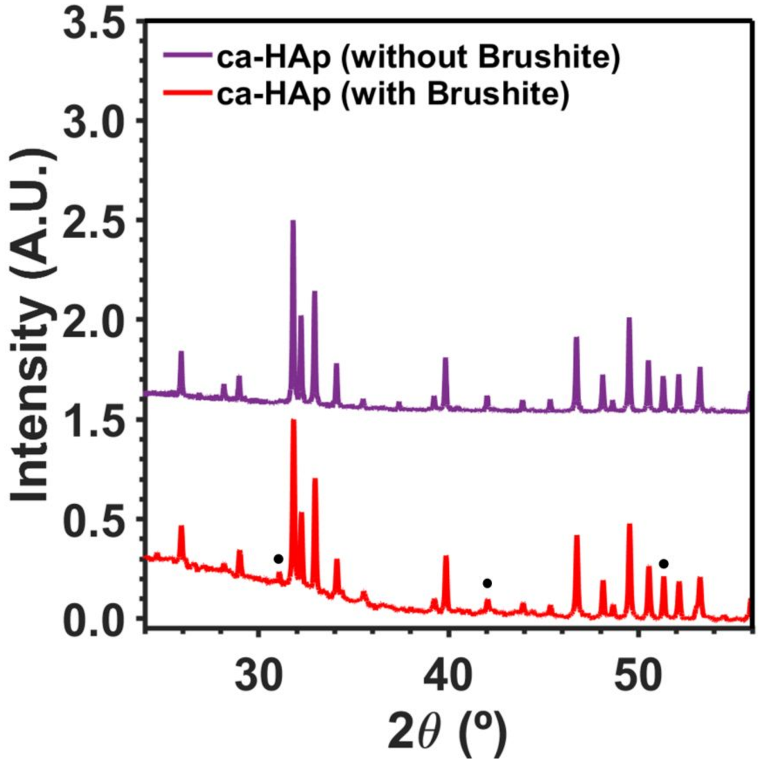

Figure S5. XRD pattern of a ca-HAp with and without Brushite. The control on the amount of Brushite obtained in the TSP treatment has been studied elsewhere.[S1] The most important diffraction peaks of Burshite are marked with black circles. As it can be seen, most of the peaks overlap with the HAp ones, hindering the proper determination of the HAp/Brushite content by means of the XRD technique.

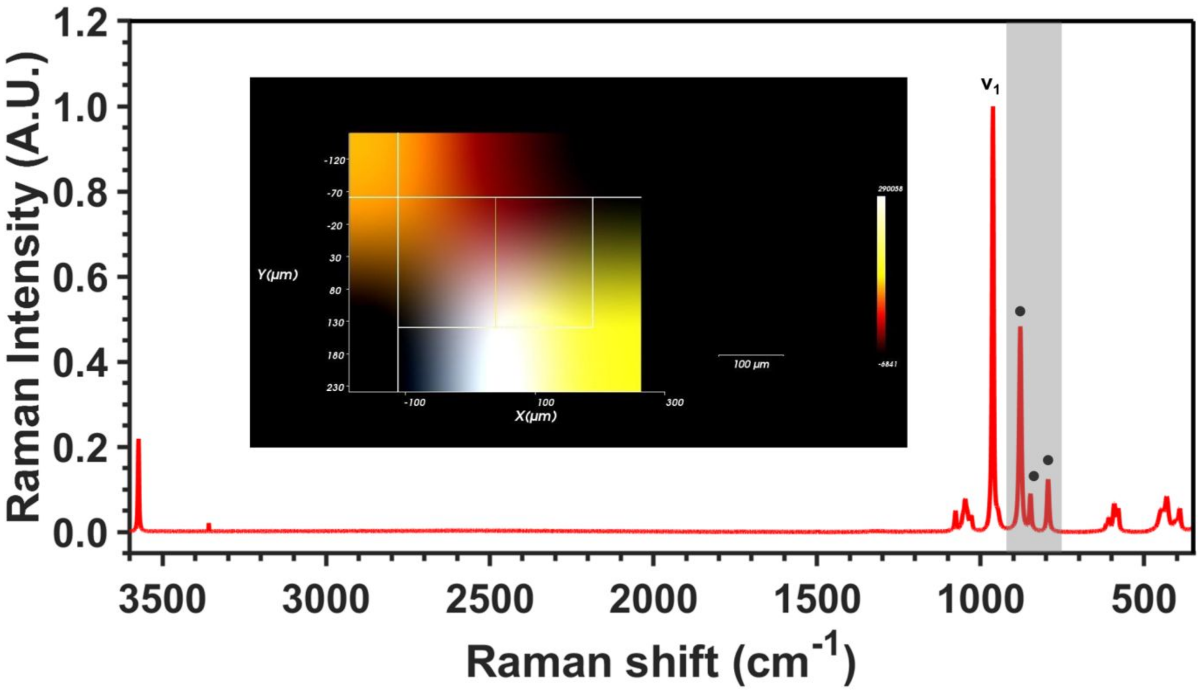

Figure S6. Raman spectrum of the ca-HAp sample. The clear peaks of Brushite at 878, 848 and 794 cm^{-1} attributed to the HPO_4^{2-} and P–OH vibrations can be observed (circles). The inset depicts a Raman map obtained from comparing the intensity 878 cm^{-1} Brushite peak with the intensity of the HAp ν_1 vibration peak. Accordingly, although the surface of the ca-HAp sample is heterogeneous, it is still dominated by HAp.

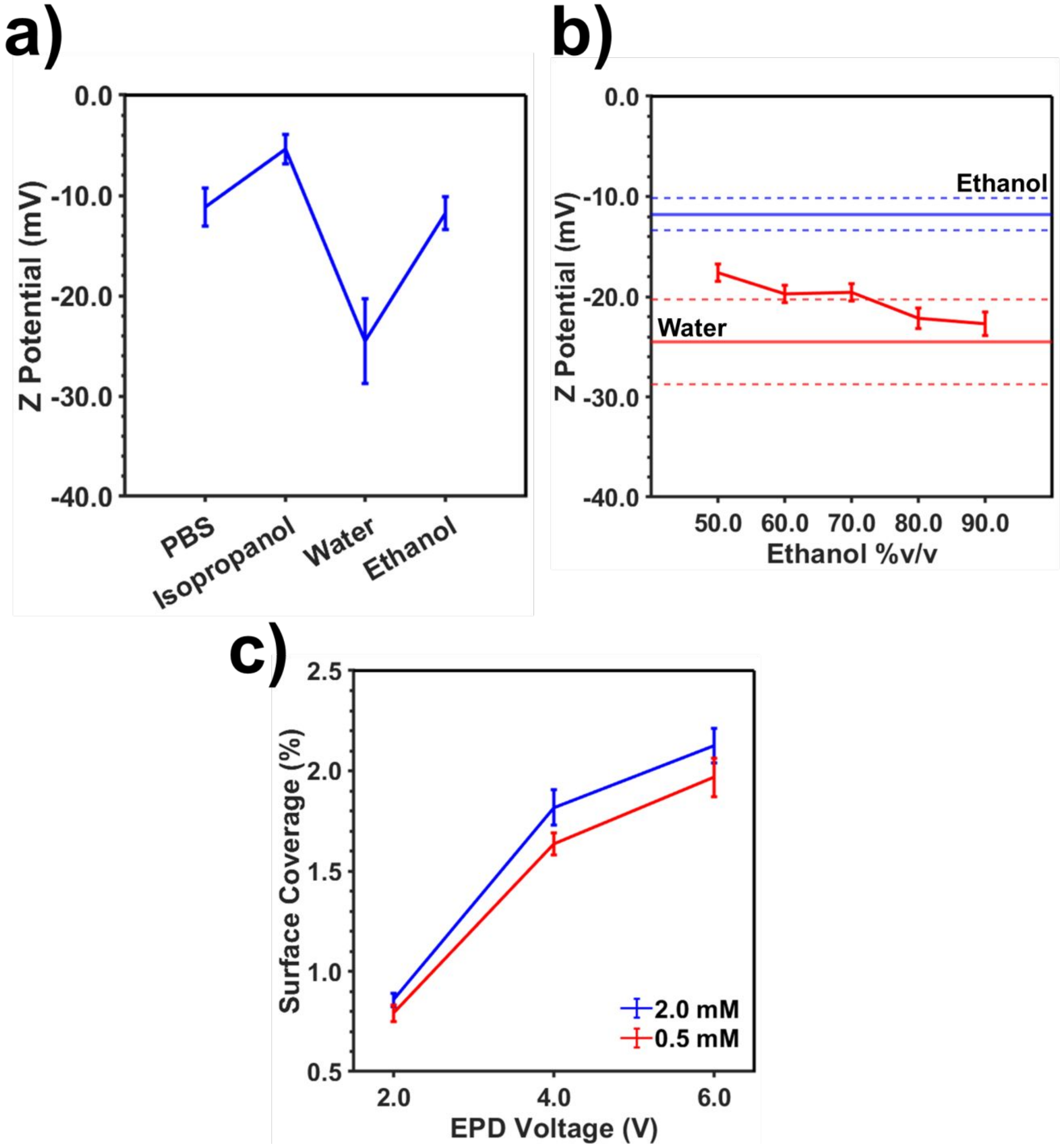

Figure S7. Optimization studies for the EPD. (a) Z-potential for ZrO_2 NPs in different solvents. Water was discarded since the results displayed high instability. (b) Z-Potential for the NPs with increasing % v/v of ethanol (water as counter solvent). Maximum absolute value for Z-Potential was obtained at 90/10% v/v ethanol:water mixture. (c) EPD voltage optimization versus the HAp surface coverage %.

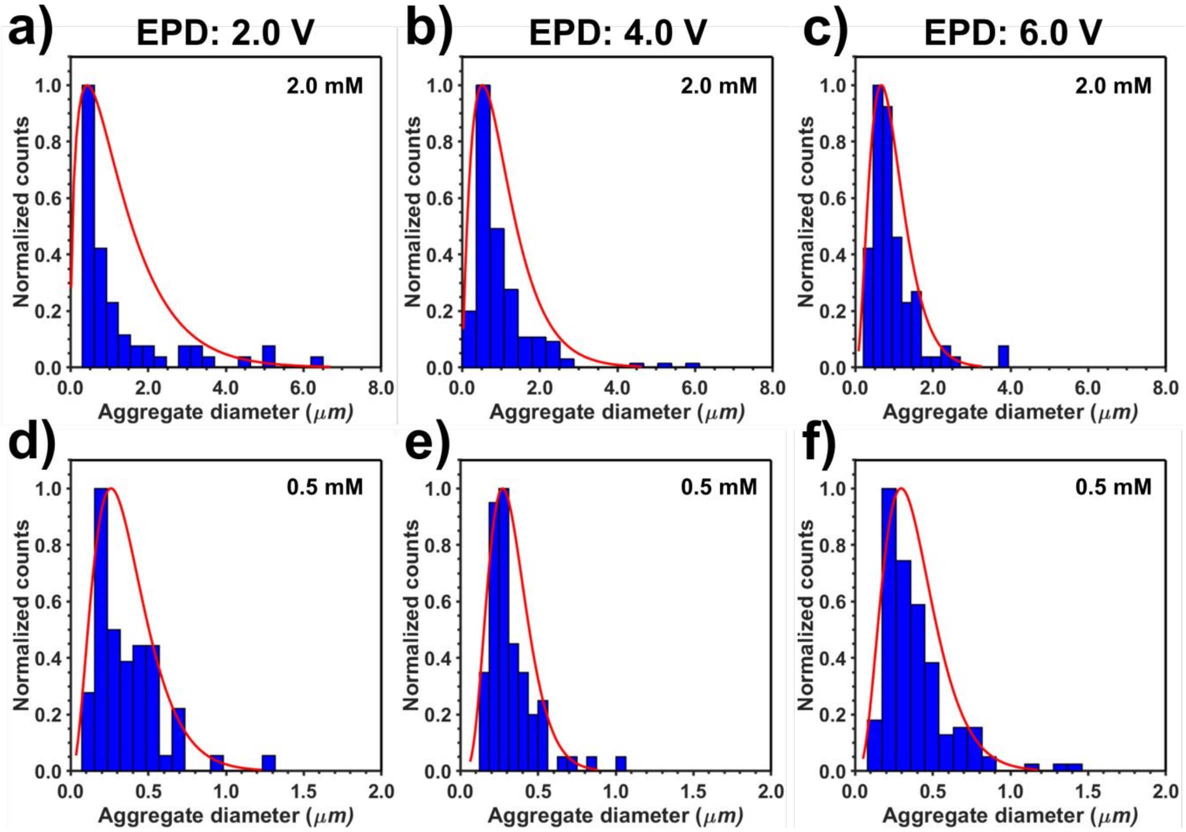

Figure S8. (a-c) Aggregate size distribution for 2.0 mM concentration of ZrO_2 at different electrophoretic deposition (EPD) voltages. (d-f) aggregate size distribution for 0.5 mM concentration of ZrO_2 at different EPD voltages.

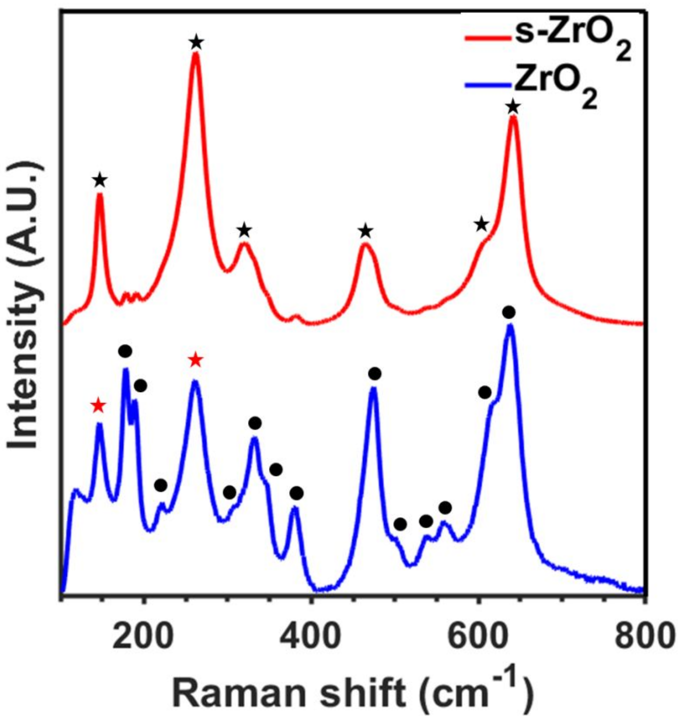

Figure S9. Raman spectra of the commercial yttrium stabilized ZrO_2. As it can be seen, after exposing ZrO_2 to the sintering treatment (s-ZrO_2; T = 1000 ºC) the tetragonal is obtained. Note that this crystallographic phase is maintained at room temperature, and thus, this polymorph is assumed for the HRTEM analyses. Peak assignation for tetragonal s-ZrO_2 (stars): 146 cm^{-1} (Zr ions movement), 261 cm^{-1} (Zr ions movement), 317 cm^{-1} (partially symmetric coupling of A_1 modes (B_{1g})), 464 cm^{-1} (partially coupling of B_1 and B_2 modes (E_g)), 609 cm^{-1} ((symmetric Zr–O–Zr stretching arising from fully symmetric coupling of A_1 modes (A_{1g})) and 640 cm^{-1} (asymmetric Zr–O–Zr stretching from fully symmetric coupling of B_1 and B_2 modes (E_g)).[S2] Peak assignation for monoclinc ZrO_2 (dots): 178 cm^{-1} (Zr–Zr; B_g+A_g vibration mode), 190 cm^{-1} (Zr–Zr), 221 cm^{-1} (Zr–Zr; B_g vibration mode), 308 cm^{-1} (Zr–O; B_g vibration mode), 332 cm^{-1}(Zr–Zr; B_g vibration mode), 347 cm^{-1} (Zr–O; A_g+B_g vibration mode), 381 cm^{-1} (Zr–O; A_g+B_g vibration mode), 475 cm^{-1} (O–O; A_g vibration mode), 502 cm^{-1} (O–O; A_g vibration mode), 537 cm^{-1} (O–O; A_g vibration mode), 557 cm^{-1} (O–O; A_g vibration mode), 615 cm^{-1} (O–O; A_g vibration mode), 638 cm^{-1} (O–O; A_g vibration mode).[S2,S3]

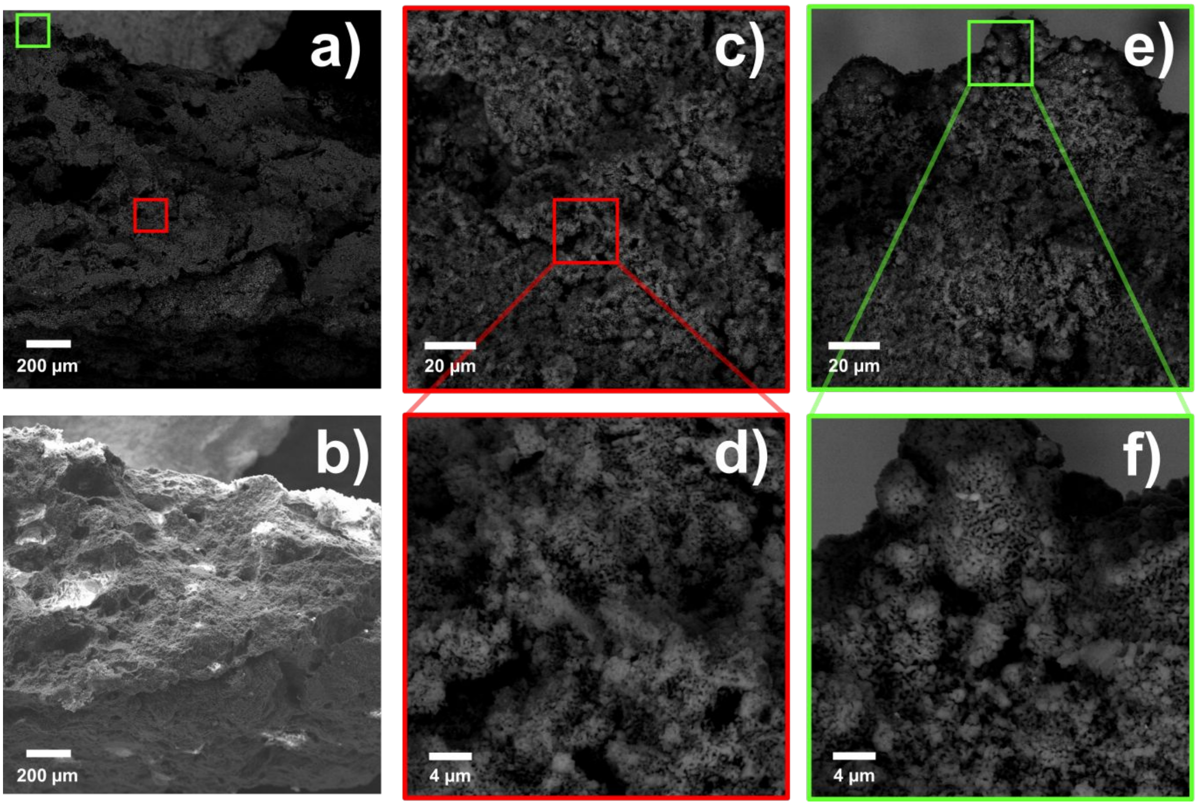

Figure S10. SEM characterization of the ca-HAp/ZrO_2 cross-section using backscattering (a) and secondary electron (b) sensors. (c-f) higher-magnification insets of (a). As it can be seen, the presence of ZrO_2 NPs in the bulk is discarded.

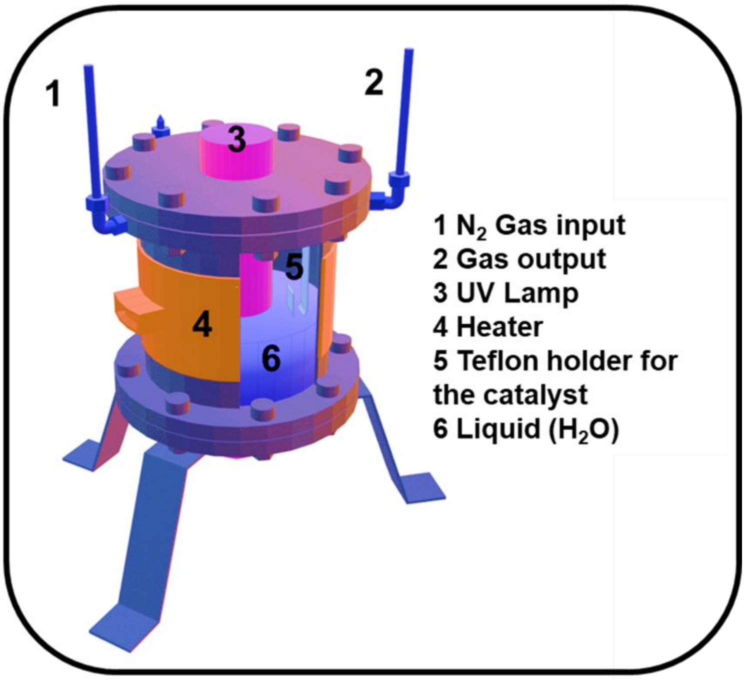

Figure S11. Scheme of the Batch reactor used.

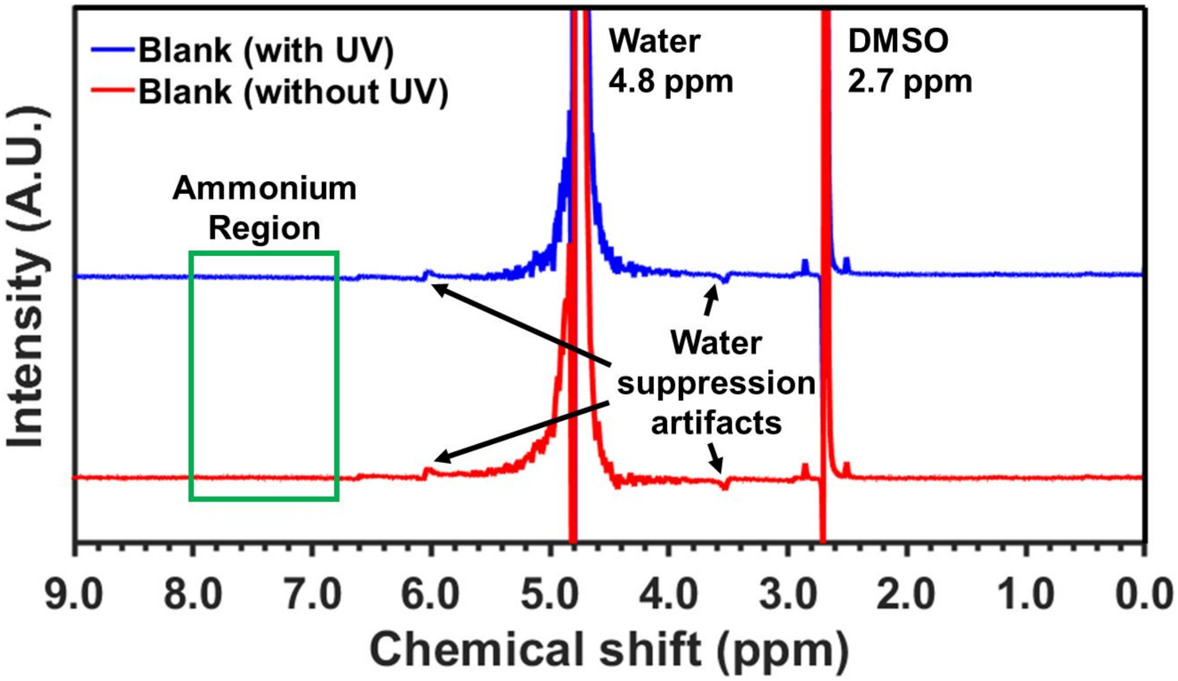

Figure S12. ^{1}H-NMR spectra of the blank reactions carried out at 120 ºC, 6 bar N_2, with 20 mL of water and for 72 hours. Without the presence of a catalyst, no ammonium is detected.

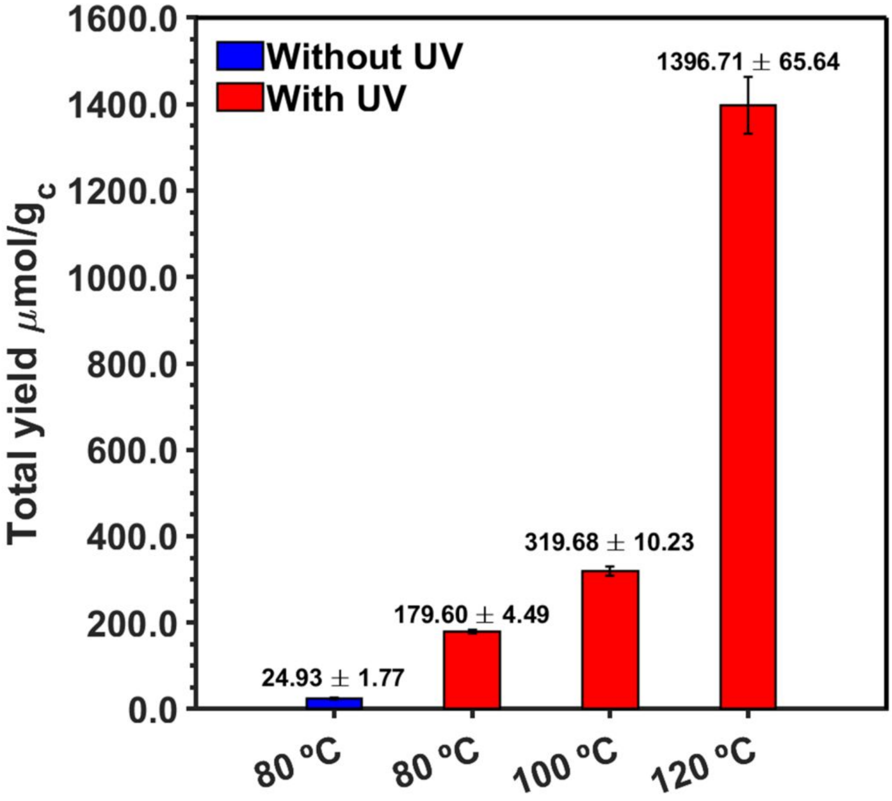

Figure S13. Reaction yield dependence on the temperature. Note that decreasing the temperature without UV irradiation also reduces the ammonia production, thus discarding other mechanisms and/or contamination sources

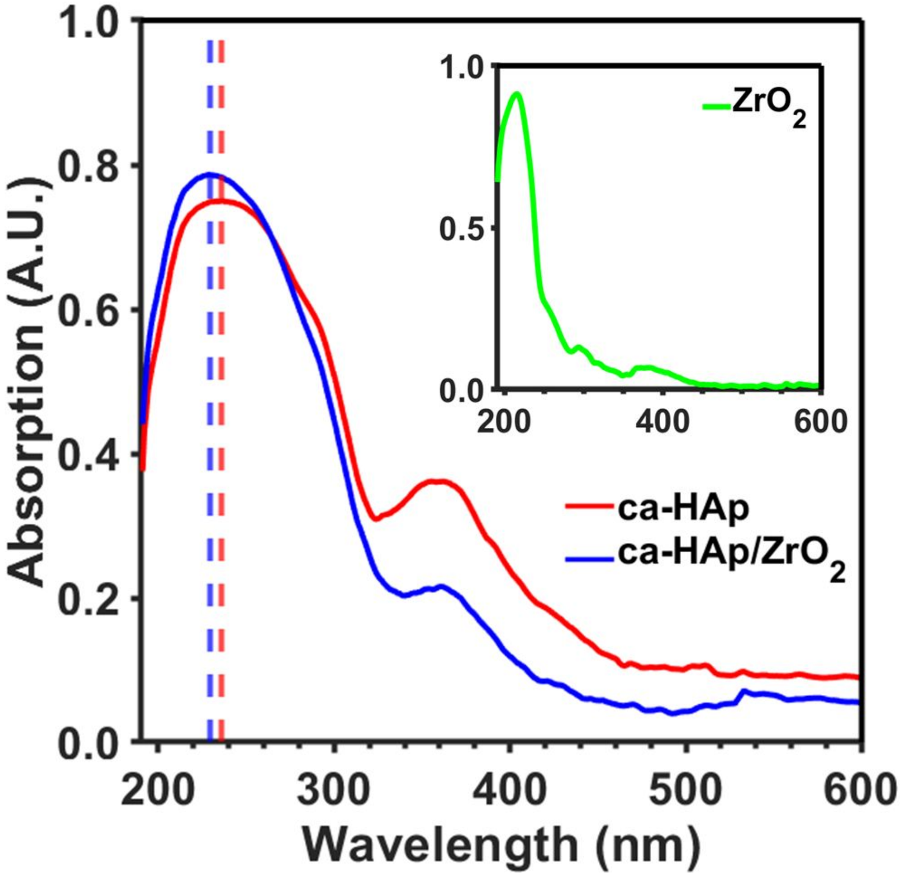

Figure S14. UV-vis spectra acquired for the samples ca-HAp, ca-HAp/ZrO_2 and ZrO_2 (inset).

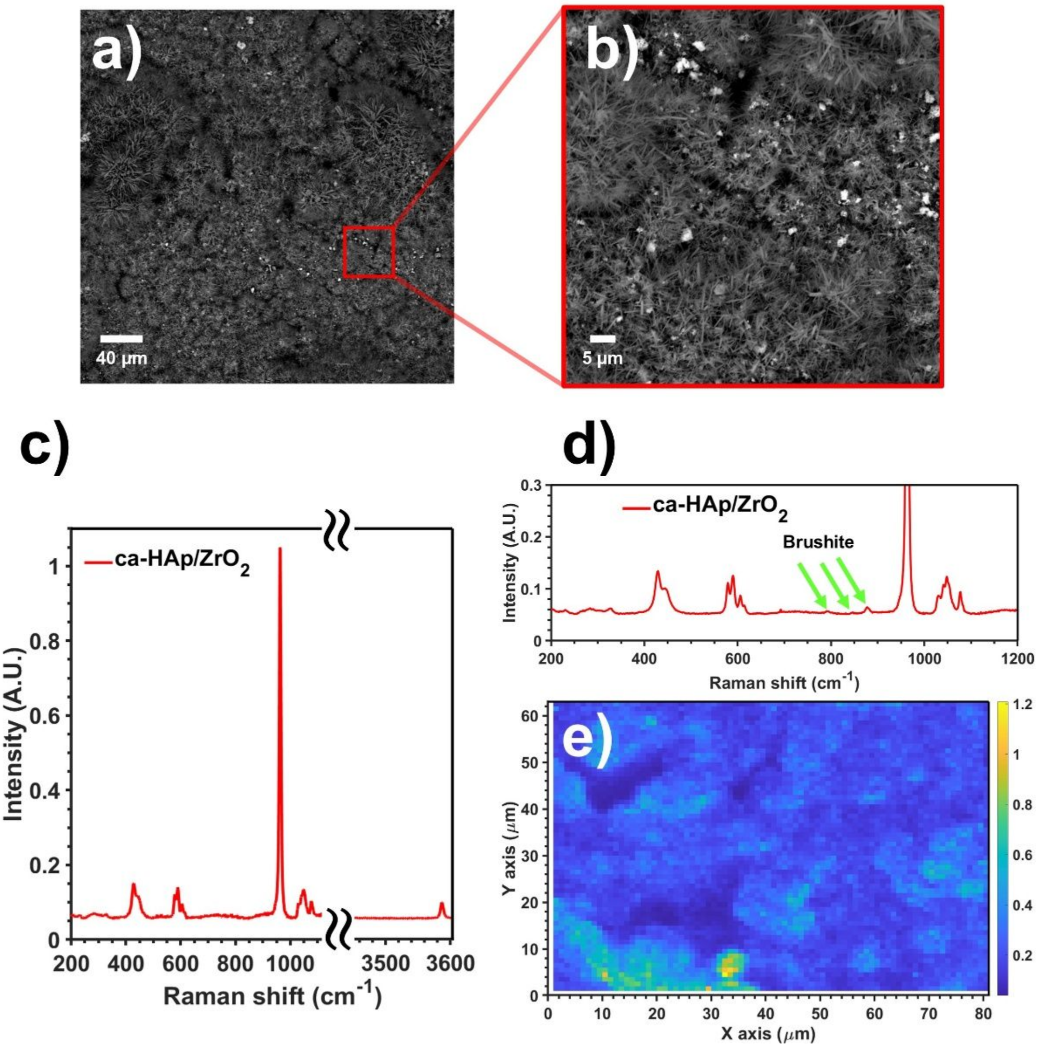

Figure S15. Structural characterization studies to determine the structural stability of the nanocomposite after the catalytic reactions. (a-b) SEM images confirming the presence and proper immobilization of ZrO_2 aggregates. A slight refinement of the ca-HAp structure into rod-like shape can be also observed, being in agreement with the literature.[S4] (c-e) Raman structural studies showing that the characteristic structure finger print of ca-HAp (*i.e.* heterogeneous presence of Bru phase is maintained after the reactions. Such result is also supported by the literature as it has been reported ca-HAp is stable up to 600-700 ºC.[S4]

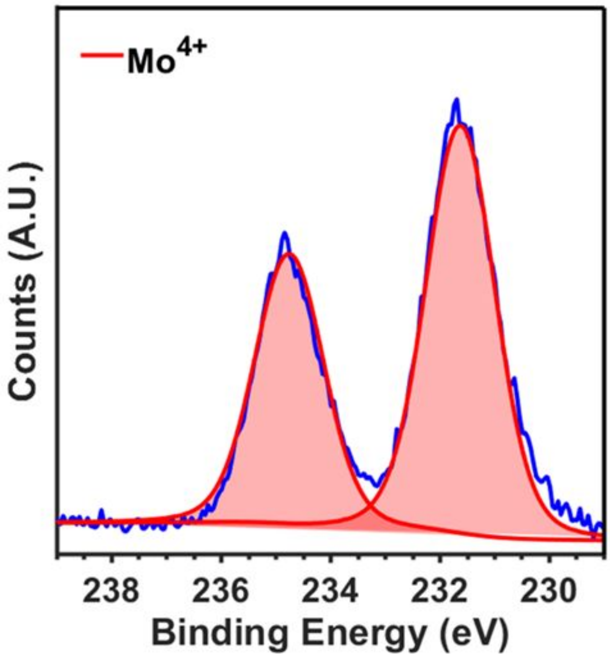

Figure S16. High-resolution spectra for the Mo 3d acquired at UHV and 120 ºC. Although the signal quality was poor due to the low amount of contamination, peak fitting (see fitting procedure in the Methods section) was achieved allowing to attribute the data to Mo^{4+}, thus indicating the presence of MoO_2 contamination (231.6, eV).[S5] Overall, we are able to state that these traces were not affecting the present study since MoO_2 is not catalytically active. In this sense MoO_3 is the main oxide used for catalysis, being used as a support for nanoparticles, with the proper vacancy engineering preparation.[S6-S8]

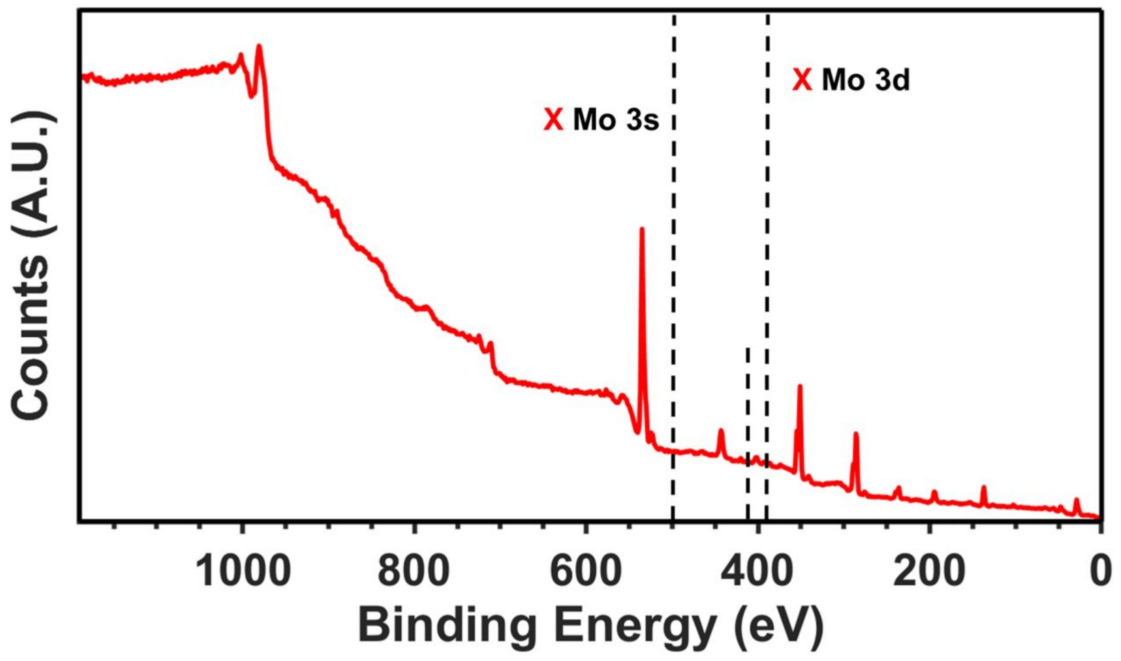

Figure S17. XPS survey of ca-HAp catalyst prepared avoiding Mo contamination. As it can be seen, the spectral lines of Mo are not detected.

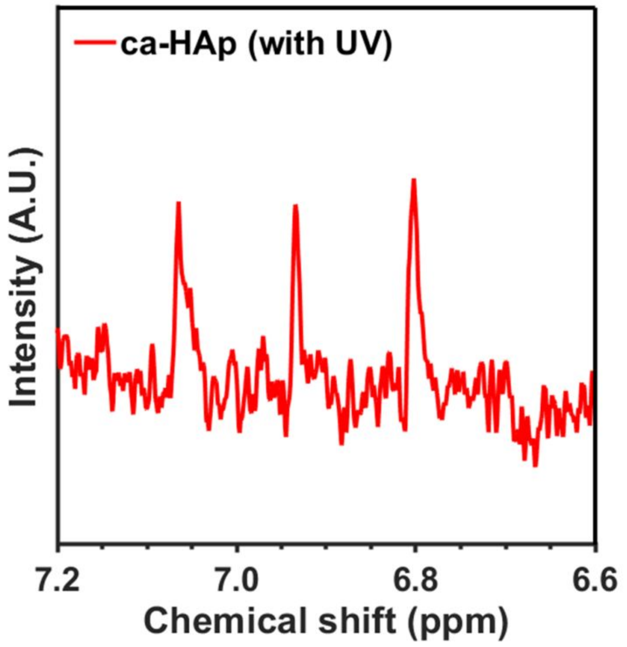

Figure S18. ^{1}H-NMR spectra obtained after a nitrogen fixation reaction (120 ºC, 6 bar N_2, 20 mL H_2O for 72 hours). Ammonium with similar yields is obtained for a ca-HAp catalyst prepared with and without the Mo contamination source, discarding any effect of such contamination.

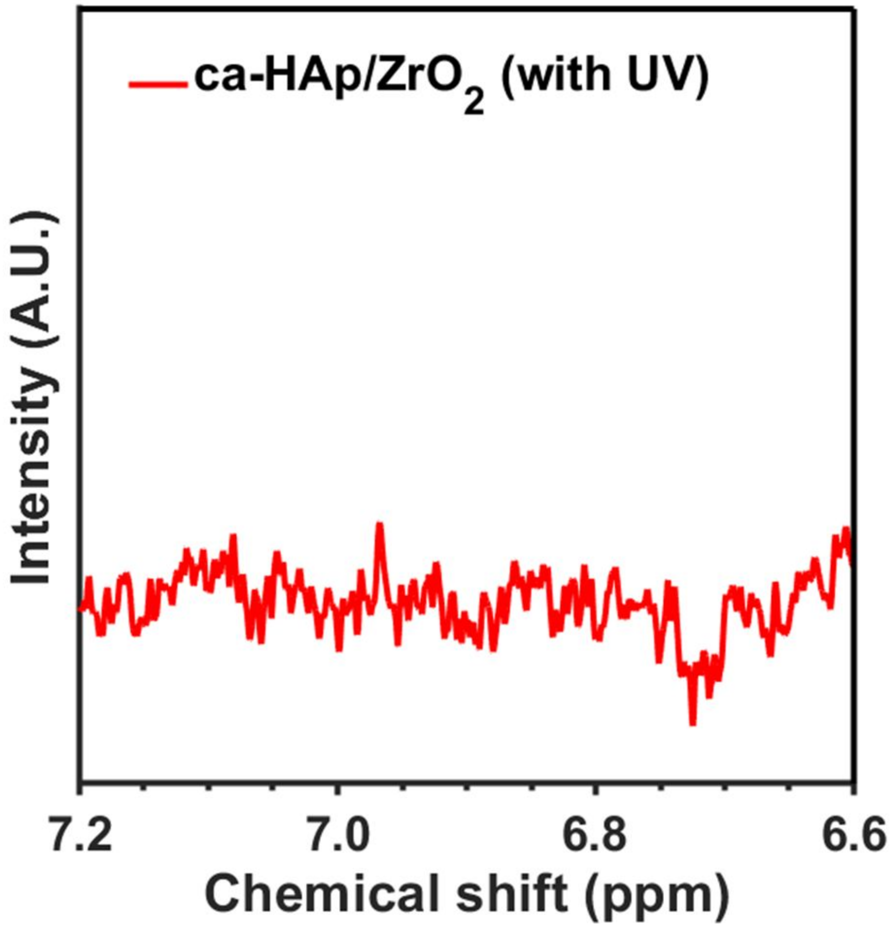

Figure S19. ^{1}H-NMR spectra obtained for dissolved ca-HAp/ZrO_2 after a nitrogen fixation reaction, where no ammonium product is observed. Thus, 100 % of NH_4^+ is found desorbed from the catalyst.

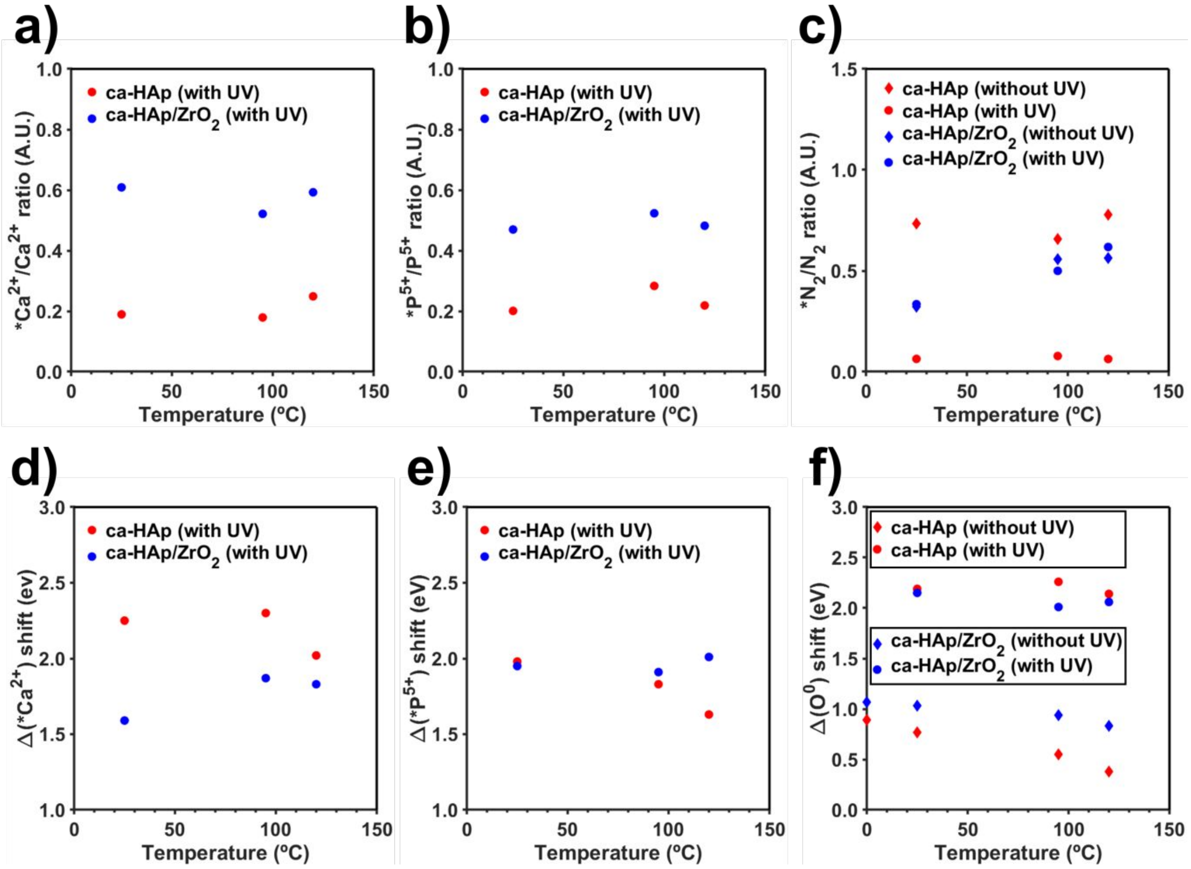

Figure S20. NAP-XPS studies performed at 25, 95 and 120 ºC for the ca-HAp and ca-HAp/ZrO_2 samples with and without UV: (a-c) Ratios of adsorbed sites and activated species for (a) Ca, (b) P and (c) N, respectively; (d-f) display the peak shift difference between adsorbed site species corresponding to (d) Ca, (e) P and (f) O, respectively.

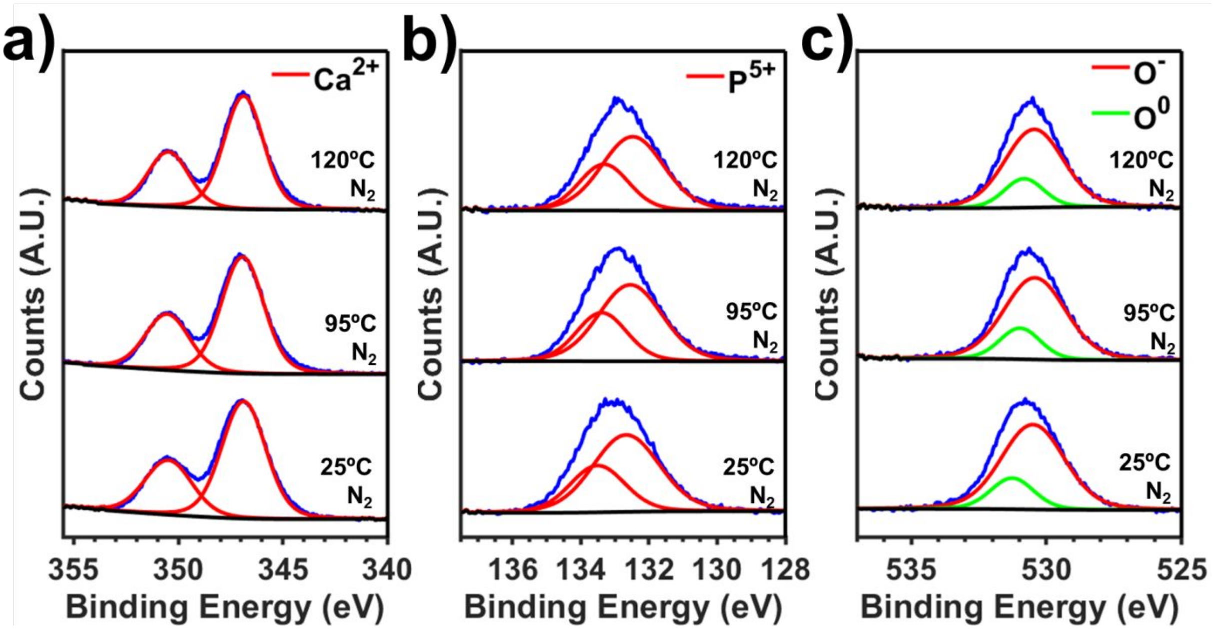

Figure S21. NAP-XPS spectra used for the studies presented in Figure S16. (a) Ca 2p, (b) P 2p and (c) O 1s regions acquired for ca-HAp at 1.0 mbar of N_2, at different temperatures and without UV irradiation.

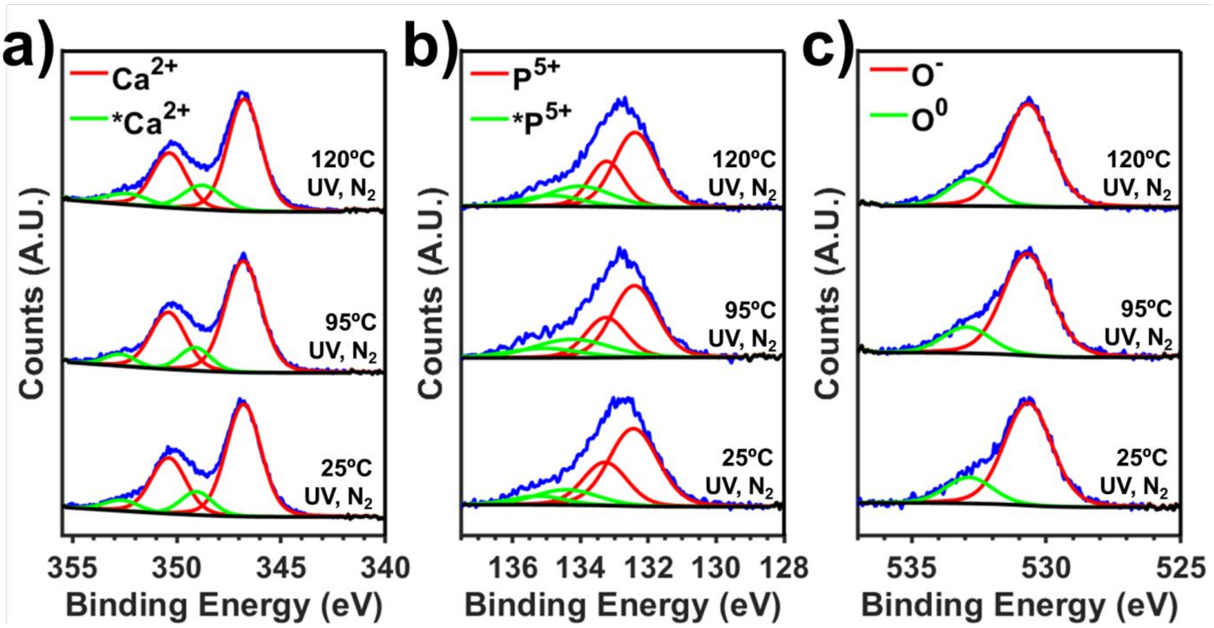

Figure S22. NAP-XPS spectra used for the studies presented in Figure S16. (a) Ca 2p, (b) P 2p and (c) O 1s regions acquired for ca-HAp at 1.0 mbar of N_2, at different temperatures and under UV irradiation (UPS source).

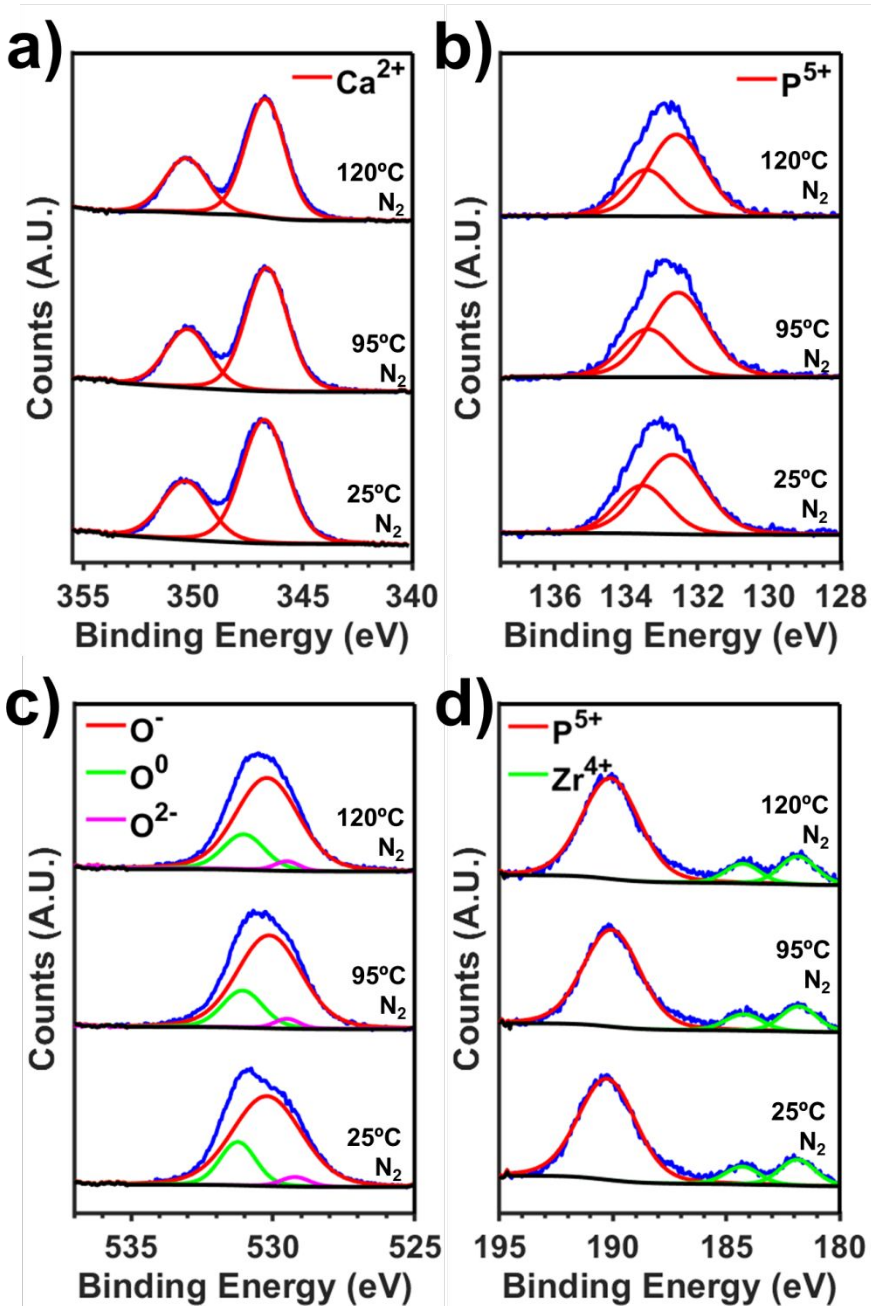

Figure S23. NAP-XPS spectra used for the studies presented in Figure S16. (a) Ca 2p, (b) P 2p, (c) O 1s and (d) P 2p and Zr 3d regions acquired for ca-HAp/ZrO_2 at 1.0 mbar of N_2, at different temperatures and without UV irradiation.

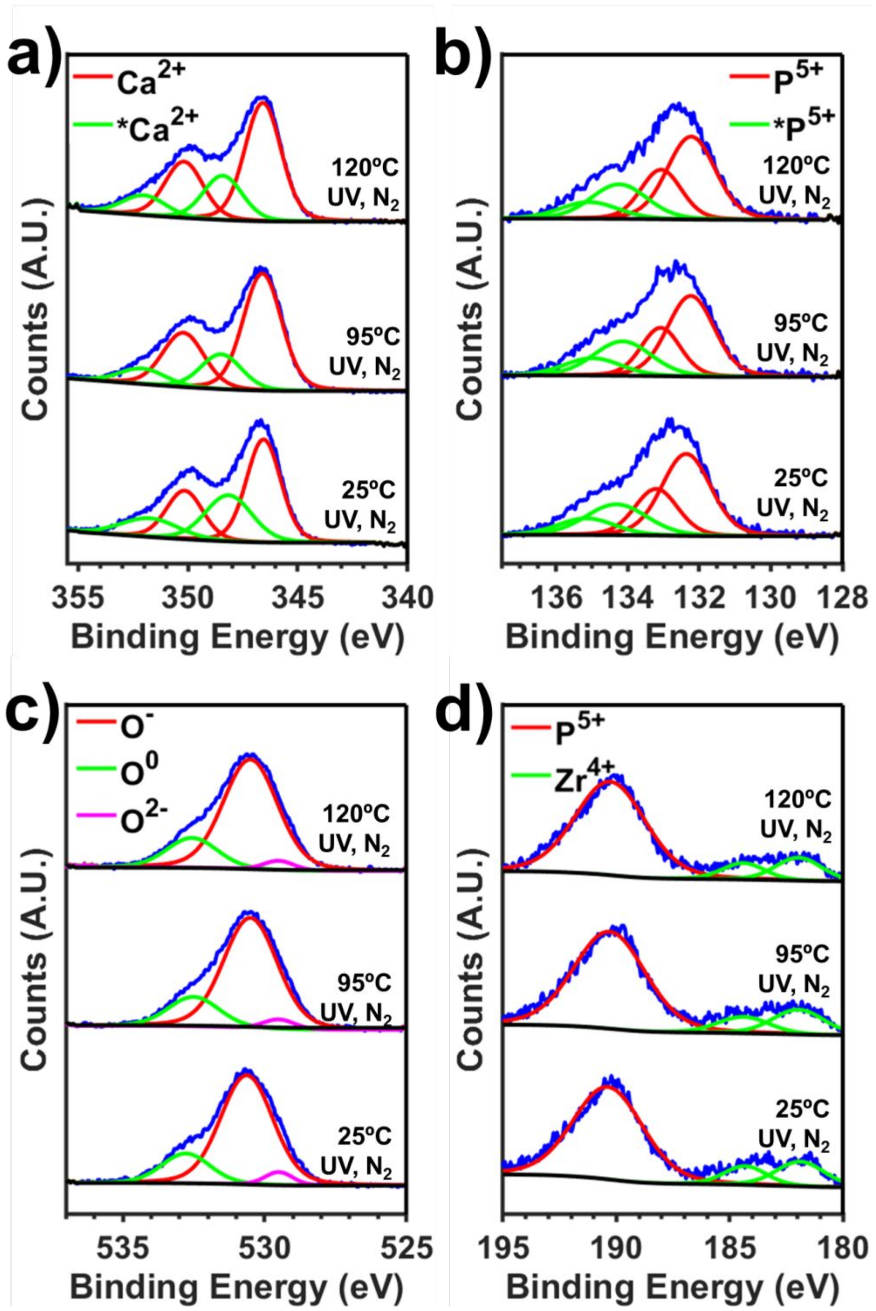

Figure S24. NAP-XPS spectra used for the studies presented in Figure S16. (a) Ca 2p, (b) P 2p, (c) O 1s and (d) P 2p and Zr 3d regions acquired for ca-HAp/ZrO_2 at 1.0 mbar of N_2, at different temperatures and under UV irradiation (UPS source).

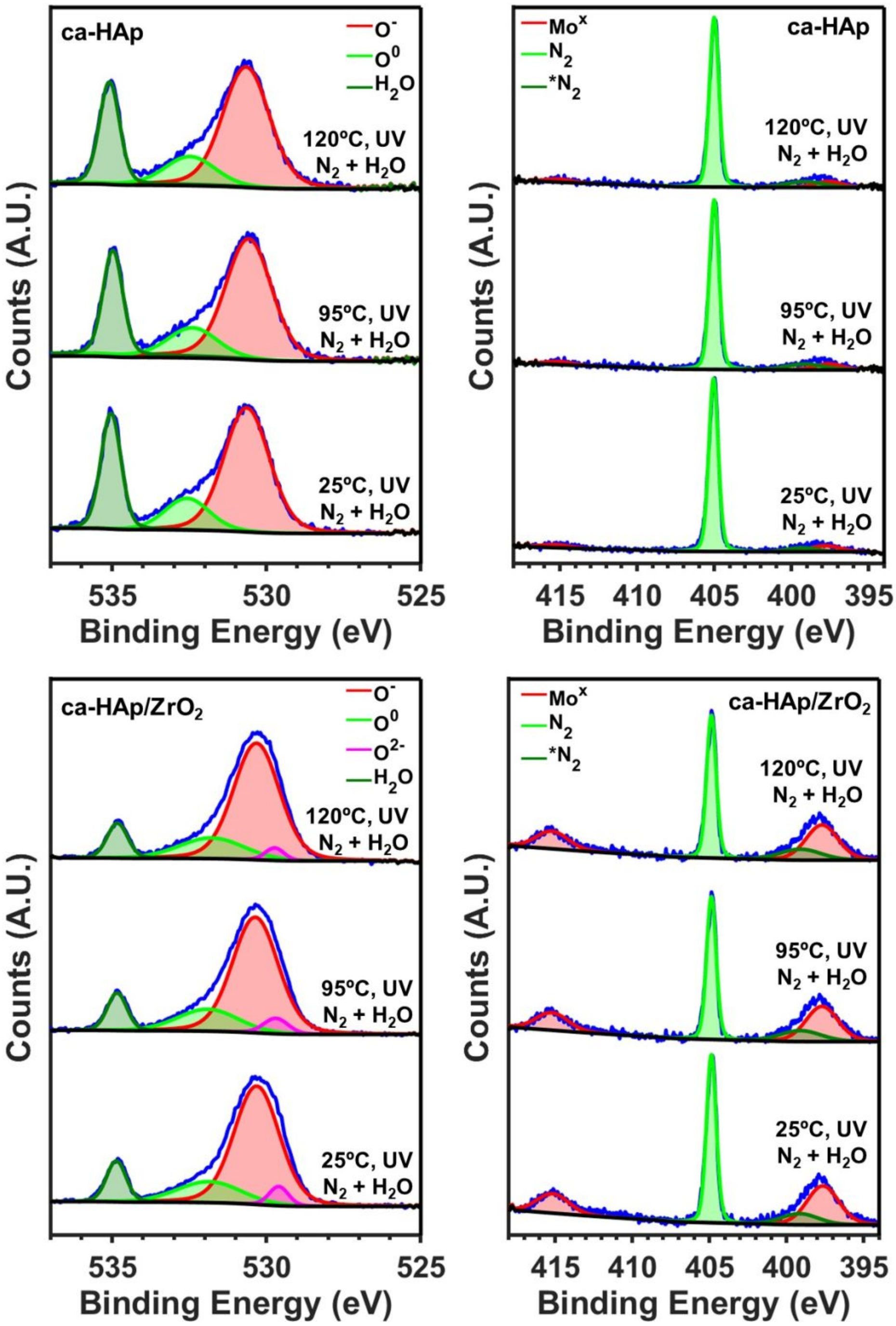

Figure S25. NAP-XPS measurements in the region of O 1s and N 1s carried out introducing also water vapor in the chamber. . It is worth highlighting the relevant increment in the number of $*N_2$ species for ca-HAp/ZrO_2 stressing out the synergy that leads to the nitrogen activation. Interestingly, the peak associated to H_2O also decreases for the ca-HAp/ZrO_2 system. Further interpretation of the experiments is complicated

due to existence of multiple reactions and phenomena associated to: (1) New XPS/UPS data acquisition challenges due to interactions with the environment of the analysis chamber; and (2) Multiple reaction steps occurring simultaneously during the measurement, including the competitive adsorption of N_2 and H_2O, their effect in the binding energies of Ca^{2+} and PO_4^{3-} sites, and the fast desorption of products (as demonstrated experimentally).

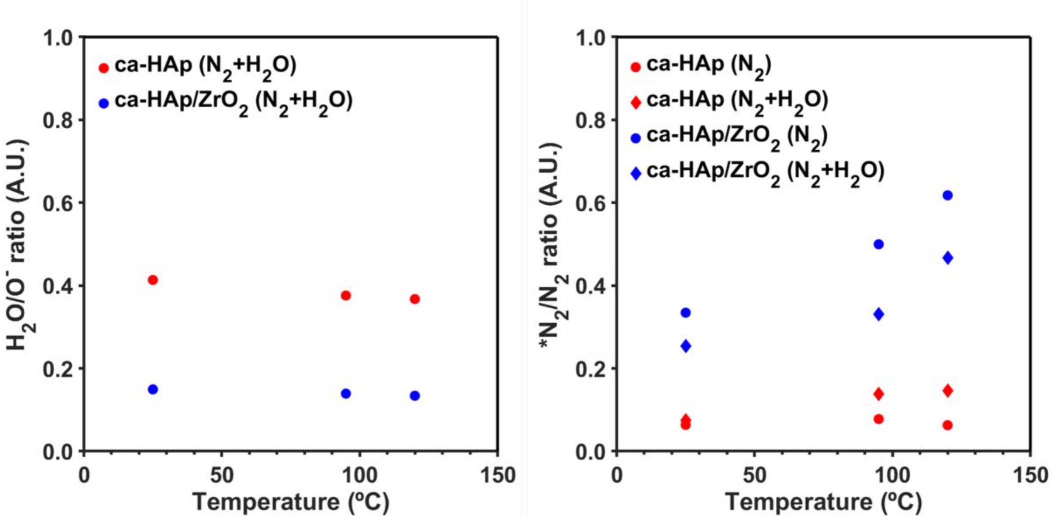

Figure S26. NAP-XPS quantifications of the H_2O/O^- ratios (left) and $*N_2/N_2$ ratios (right) derived from the N_2:H_2O measurements.

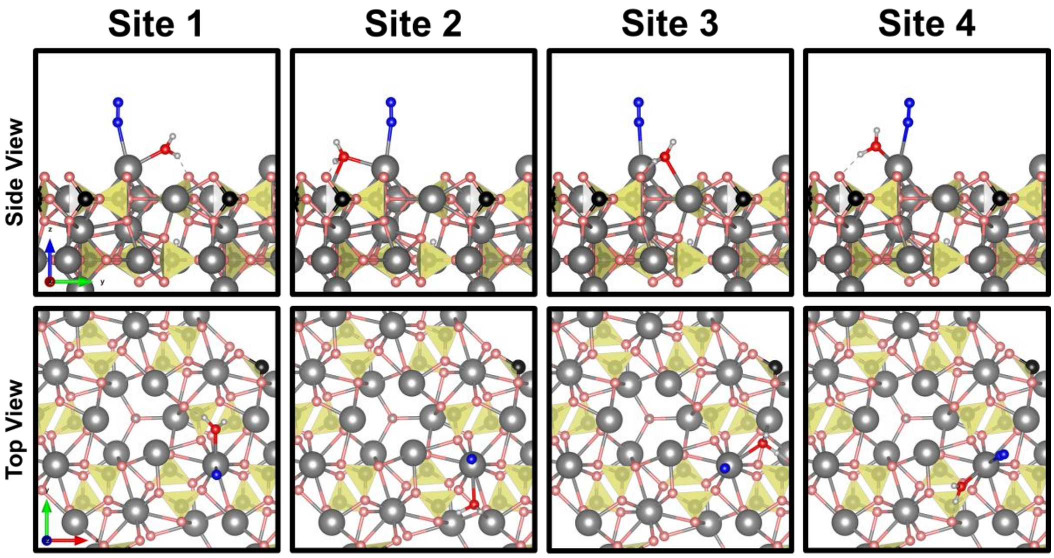

Figure S27. Adsorption energy studies for simultaneous N_2 and H_2O molecules in the binding site HAp-2.

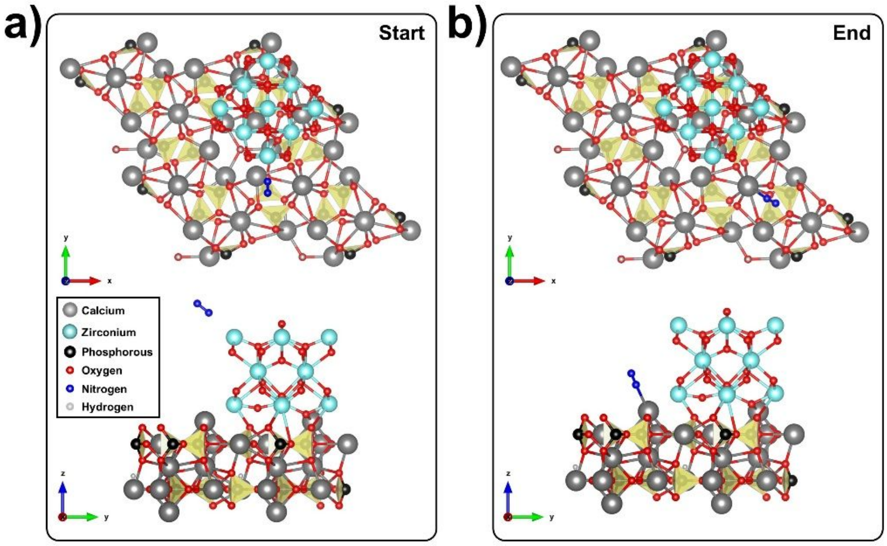

Figure S28. (a-b)) Starting and ending points used to perform the NEB calculations related to the nitrogen spill over mechanism proposed. The ZrO_2 NP has been located at the north-east crystallographic position with respect the ca-HAp lattice

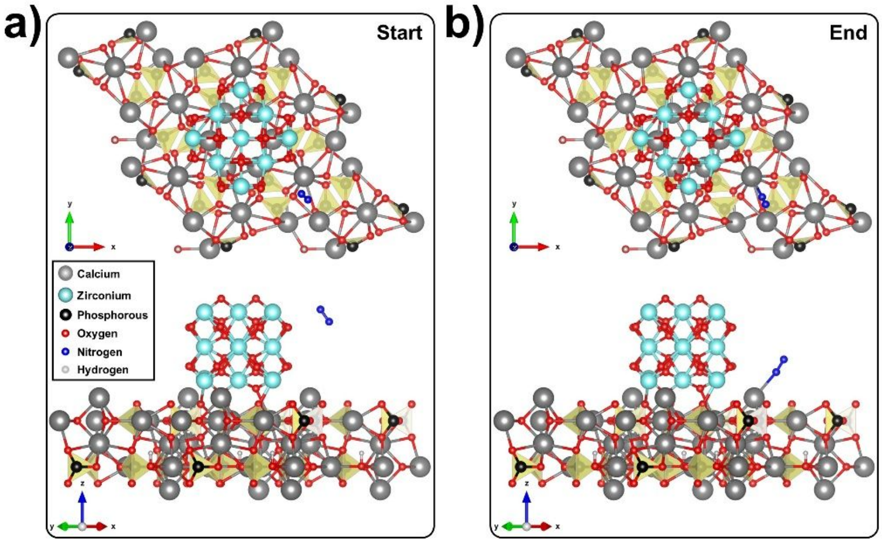

Figure S29. (a-b)) Starting and ending points used to perform the NEB calculations related to the nitrogen spill over mechanism proposed. The ZrO_2 NP has been located centered (on top the OH^- columns) with respect the ca-HAp lattice.

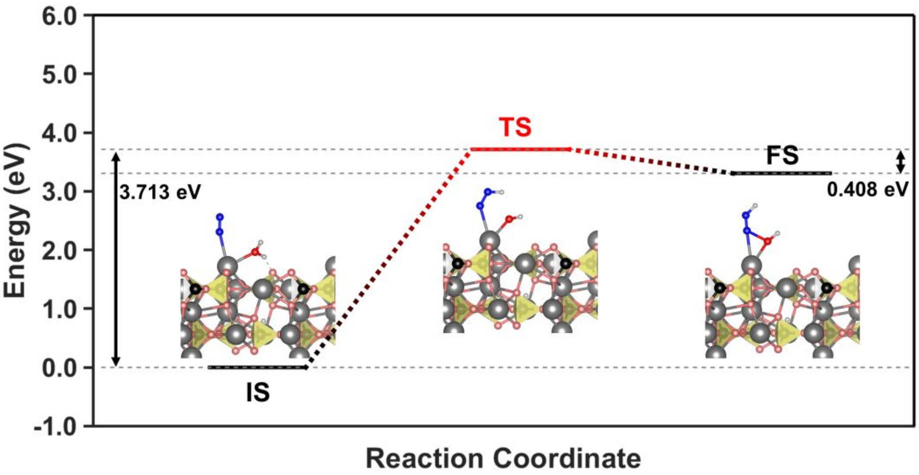

Figure S30. Energy barrier associated to the water-splitting and incorporation in intermediate

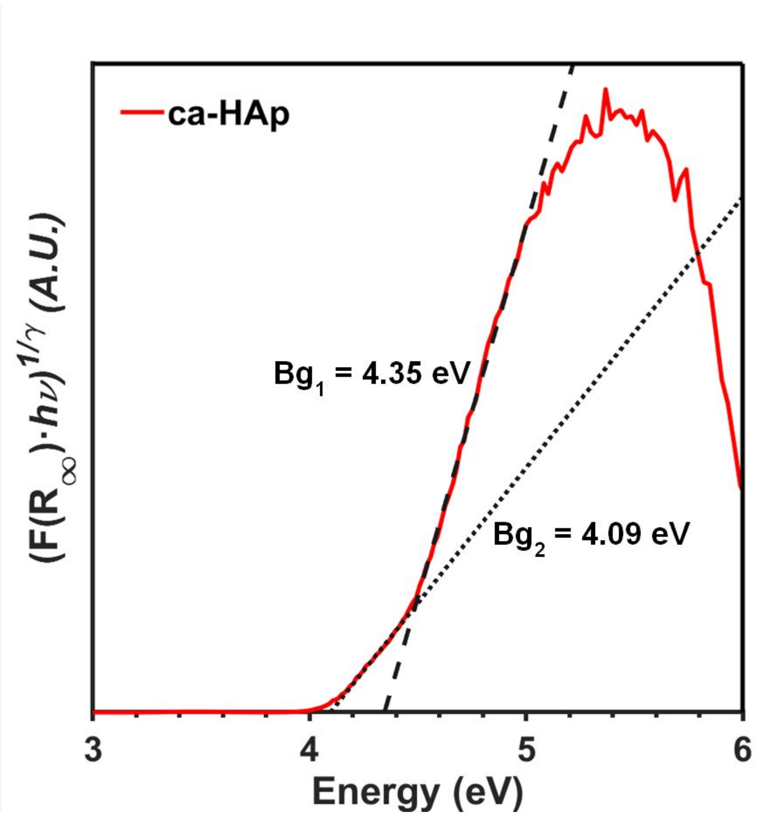

Figure S31. Tauc plot. Two regimes are observed with a band gap between 4.0-4.4 eV

REFERENCES

S1 Sans, J.; Arnau, M.; Sanz, V.; Turon, P.; Alemán, C. Polarized Hydroxyapatite: New Insights and Future Perspectives Through Systematic Electrical Characterization at the Interface. Advanced Materials Interfaces, 9, 2101631 (2022).

S2 López, E. F.; Escribano, V. S.; Panizza, M.; Carnasciali, M. M.; Busca, G. Vibrational and electronic spectroscopic properties of zirconia powders. J. Mater. Chem., 11, 1891-1897 (2001).

S3 Kim, B.-K.; Hamaguchi, H.-O. Mode Assignements of the Raman Spectrum of Monoclinic Zirconia by Isotopic Exchange Technique. Phys. Stat. Sol. (b) 203, 557-563 (2001).

S4 M. Arnau, et al. Unraveling Thermal Depolarization Phenomena in Biphasic Polarized Calcium Phosphate Catalyst. Adv. Mater. Interfaces 2024, 11, 2400422.

S5 Scanlon, D. O. et al. Theoretical and Experimental Study of the Electronic Structures of MoO_3 and MoO_2. J. Phys. Chem. C, 114, 4636-4645 (2010).

S6 Zhang, L. et al. Active Hydrogen-Switchable Dynamic Oxygen Vacancies in MoO_{3-x} upon Ru Nanoparticle Decoration for Boosting Photocatalytic Ammonia Synthesis Performance. ACS Catal. 14, 5696-5709 (2024).

S7 Han, L. et al. Selective Catalytic Reduction of NO_x with NH3 by Using Novel Catalysts: State of the Art and Future Prospects. Chem. Rev. 119, 10916 – 10976 (2019).

S8 Liu, T. et al. Engineering of local electron properties optimization in single-atom catalysts enabling sustainable photocatalytic conversion of N_2 into NH_3. Chem. Eng. J. 489, 150286 (2024).
